# A single non-coding SNP in FPGS modulates folate drug efficacy in acute lymphoblastic leukemia: data-driven exploration and experimental validation

**DOI:** 10.1186/s43556-025-00353-9

**Published:** 2025-11-21

**Authors:** Wenliang Yu, Chenyang Li, Yuning Meng, Qiang Li, Mengyue Gao, Wei Tang, Yao Li, Ziyi Tan, Xiaoran Zhou, Zeyang Liu, Yun Xu, Zichun Hua

**Affiliations:** 1 The State Key Laboratory of Pharmaceutical Biotechnology and Department of Neurology of Nanjing Drum Tower Hospital, School of Life Sciences and The Affiliated Hospital of Nanjing University Medical School, Nanjing University, Nanjing, 210023 China; 2 Changzhou High-Tech Research Institute of Nanjing University and Jiangsu TargetPharma Laboratories Inc., Changzhou, 213164 China; 3 Faculty of Pharmaceutical Sciences, Xinxiang Medical University, Xinxiang, 453002 China

**Keywords:** Folylpoly-γ-glutamate synthetase, Non-coding SNP, Genetic variants, Acute lymphoblastic leukemia, Methotrexate

## Abstract

For over 70 years, methotrexate (MTX) has remained a first-line chemotherapeutic agent for acute lymphoblastic leukemia (ALL), playing a pivotal role in maintenance therapy. Understanding the genetic determinants of MTX efficacy is therefore essential for improving clinical outcomes. However, studies on MTX efficacy–related polymorphisms remain limited, particularly for non-coding variants, for which most evidence is based on statistical associations. Here, through integrative bioinformatics analysis and systematic meta-analysis, we identified rs1544105, a non-coding SNP in the folylpoly-γ-glutamate synthetase (FPGS) gene, as closely associated with MTX efficacy. Compared with the GG genotype, the AA genotype increased disease progression risk (OR: 2.23; 95% CI: 1.16–4.30; *p* = 0.017) and elevated plasma MTX concentration-to-dose ratios at 24 h (WMD: 2.27; 95% CI: 1.04–4.40; *p* = 0.002) and 40 h (WMC: 0.02; 95% CI: 0.00–0.04; *p* = 0.033). Using prime editing, we generated homozygous mutant (GG) 293T cells, demonstrating that rs1544105 A > G increased FPGS expression (~ 1.5-fold, *p* < 0.05) and intracellular MTX retention (*p* < 0.05). Moreover, both cell-based and animal experiments confirmed that rs1544105 A > G markedly improved MTX efficacy. Mechanistically, dual-luciferase reporter and electrophoretic mobility shift assays revealed that rs1544105 A > G enhanced the binding affinity of the SNP-containing sequence for the transcription factor CREB1, thereby increasing FPGS transcriptional activity and ultimately augmenting MTX efficacy. Our multidimensional study, integrating data analysis with cellular, molecular, and animal experiments, highlights the remarkable regulatory role of a single SNP, rs1544105, in modulating MTX therapeutic response and provides a basis for individualized MTX-based maintenance therapy in ALL patients.

**Supplementary Information:**

The online version contains supplementary material available at 10.1186/s43556-025-00353-9.

## Introduction

Acute lymphoblastic leukemia (ALL) is the most common pediatric hematologic malignancy [1]. While chemotherapy achieves an initial remission rate of approximately 80% in children with ALL, chemoresistance-driven relapse remains a formidable challenge, significantly compromising long-term survival [2]. The global relapse rate of ALL remains high (15–20%), and each subsequent relapse worsens the prognosis [3]. Thus, developing personalized maintenance therapies tailored to relapsed ALL patients is imperative. Encouragingly, advances in the genomic profiling of relapsed ALL samples have improved our mechanistic understanding of chemotherapy resistance [4, 5], laying the foundation for integrating pharmacogenomics with experimental validation to optimize personalized treatment strategies.

For more than 70 years, weekly Methotrexate (MTX)-based maintenance therapy has played a crucial role in achieving durable remission and potential cure in ALL [6–8]. As a folate antagonist [9], MTX inhibits tumor cell folate metabolism, thereby disrupting DNA replication and other essential cellular processes, and ultimately exerting antitumor effects [10–12]. However, owing to its nature as a divalent anion [13], the transmembrane transport of MTX relies on active transport mediated by specific membrane transporters and receptors, such as proton-coupled folate transporters, reduced folate carriers, and folate receptors [14, 15]. Moreover, MTX is highly susceptible to recognition and efflux by the bidirectional anion transporter reduced folate carrier and ATP-driven efflux pumps, which limit its intracellular retention and therapeutic effectiveness. Thus, enhancing intracellular MTX accumulation is critical for maximizing its therapeutic potential.

Folylpoly-γ-glutamate synthetase (FPGS) plays a pivotal role in prolonging intracellular MTX retention. It exists in two isoforms: cytosolic FPGS, which is involved in nucleotide biosynthesis, and mitochondrial FPGS, which is involved in glycine metabolism [16–18]. Once MTX enters the cell, FPGS rapidly catalyzes the addition of multiple glutamate residues to its γ-carboxyl group, converting MTX into a polyanion [19, 20]. This process significantly enhances its intracellular retention while also increasing the affinity of MTX for its target enzymes, such as thymidylate synthase [21, 22]. Therefore, genetic polymorphisms or mutations at FPGS-related loci represent potential key factors in ensuring the efficacy of MTX maintenance therapy.

In recent years, the CRISPR-Cas9 genome editing toolbox has rapidly evolved [23]. Among these advancements, prime editing (PE)—pioneered by Liu’s team—has revolutionized the precise modelling of drug-genome interactions [24]. This technology uses a dual-plasmid system to simultaneously deliver fusion-expressed Cas9n protein and reverse transcriptase, along with engineered RNA containing the target mutation sequence, into the cells to be edited. Single-strand breaks are induced near the target editing site and the subsequent repair processes incorporate the mutation into the genome. PE offers a powerful editing tool for functional validation of pharmacogenomic variants.

There are many single nucleotide polymorphism (SNP) loci in both the coding and non-coding regions of the FPGS gene. Previous studies have identified several relevant loci in the coding regions. These loci directly affect the amino acid sequence or structure of the FPGS protein [25]. In contrast, most studies of SNP loci in the non-coding regions have only used statistical analyses without experimental validation. And the findings from these studies remain controversial. For example, Liu et al. [26] and Giletti et al. [27] reported that FPGS rs1544105 polymorphisms may affect MTX efficacy and adverse events, whereas Muralidharan et al. [28] found no association between FPGS rs1544105 polymorphisms and MTX treatment outcomes. Therefore, this study not only identified SNPs most relevant to MTX maintenance therapy through bioinformatics and systematic meta-analysis, but also generated corresponding mutant cell lines using PE. Building on this, a series of molecular, cellular, and animal experiments were performed to validate the results and investigate the underlying mechanisms. Given that FPGS rs1544105 G > A is a highly frequent variant (allele frequency = 0.483, gnomAD v4.1.0 dataset) [29], these findings have significant translational potential for advancing precision medicine in MTX-based therapy and improving clinical outcomes for ALL patients.

## Results

### FPGS gene deletion and SNP mutations as key factors in ALL relapse

**Fig. 1:**
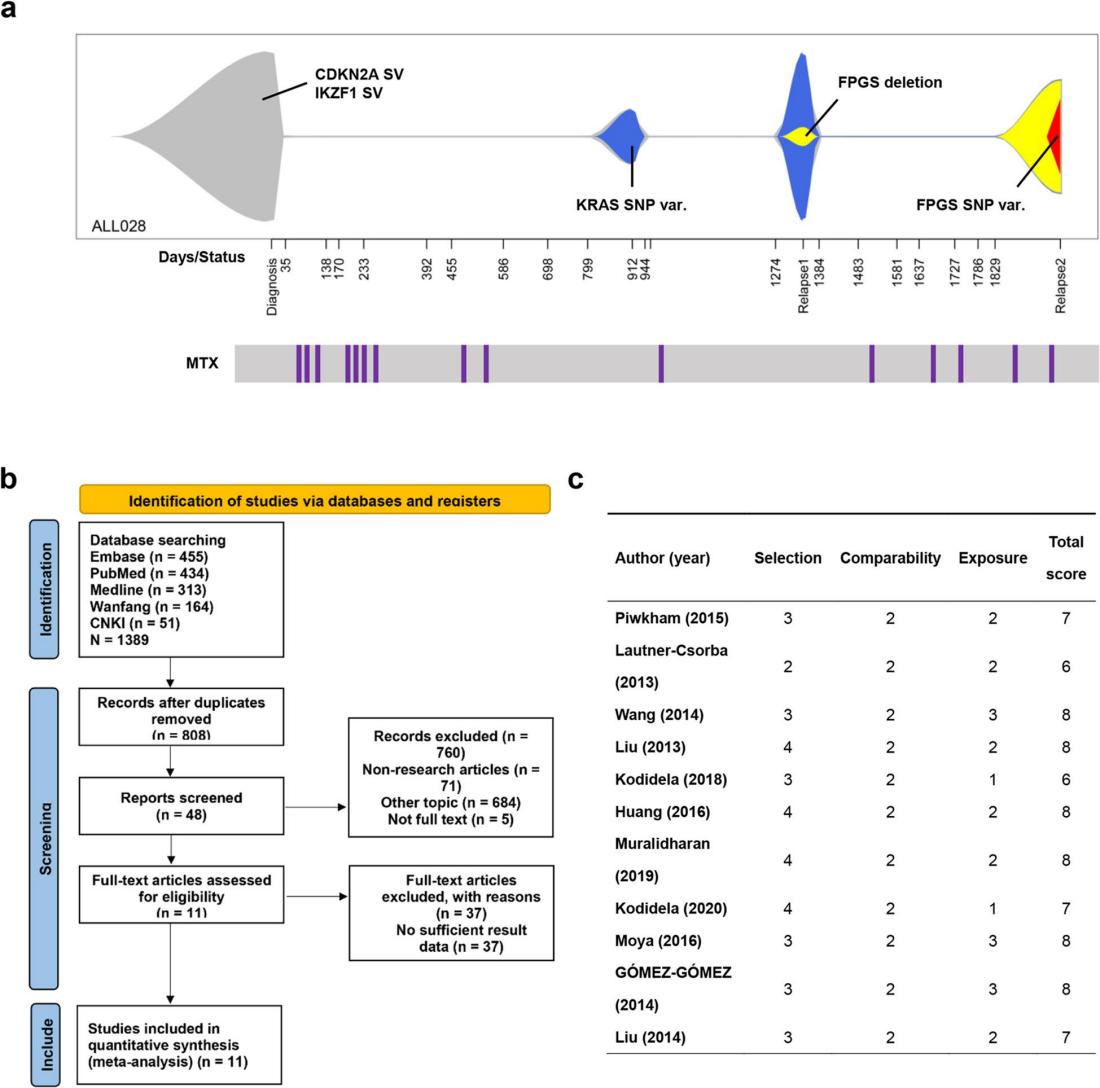
Bioinformatics analysis and systematic meta-analysis workflow. **a** Clonal evolution fish plot of a relapsed ALL patient (ALL028). **b** PRISMA flowchart depicting the study selection process for the systematic meta-analysis. **c** Quality assessment of the included studies in a systematic meta-analysis. In panel (**a**), the X-axis represents the number of days from diagnosis, with relapse events labeled as Relapse1 and Relapse2. The Y-axis denotes the relative clone size, with distinct colors representing different genetic clones, as indicated in the legend below each plot. MTX treatment time points are annotated beneath the fish plot

To explore the impact of FPGS gene mutations on MTX maintenance therapy, bioinformatics analysis was performed on a reported ALL patient (ALL028) who relapsed twice during MTX maintenance (at months 45 and 66). The first relapse involved a focal FPGS deletion (~ 20% clonality), and the second showed a mutation at the FPGS SNP locus (> 60% clonality) (Fig. 1a). These findings indicate that specific SNP mutations in the FPGS gene may contribute to disease relapse in ALL patients receiving MTX maintenance therapy.

### FPGS rs1544105 polymorphism significantly associates with ALL progression and MTX treatment outcomes

To investigate the association between FPGS SNP polymorphisms and ALL progression as well as MTX efficacy, we conducted a systematic meta-analysis. The literature selection process is depicted in the Preferred Reporting Items for Systematic Reviews and Meta-Analyses (PRISMA) flow diagram (Fig. 1b). After screening 1,389 publications, 11 studies were included. all the studies included in the analysis had NOS scores greater than 5 (Fig. 1c), and the genotype frequencies in the control groups were consistent with the Hardy–Weinberg equilibrium (HWE). No studies were excluded due to deviations from HWE. Supplementary Table 2 presents the SNP data extracted from each study for analysis, including three FPGS gene-related SNP sites located in the promoter (rs1544105), exon (rs10105), and enhancer (rs10760502) regions.

**Fig. 2:**
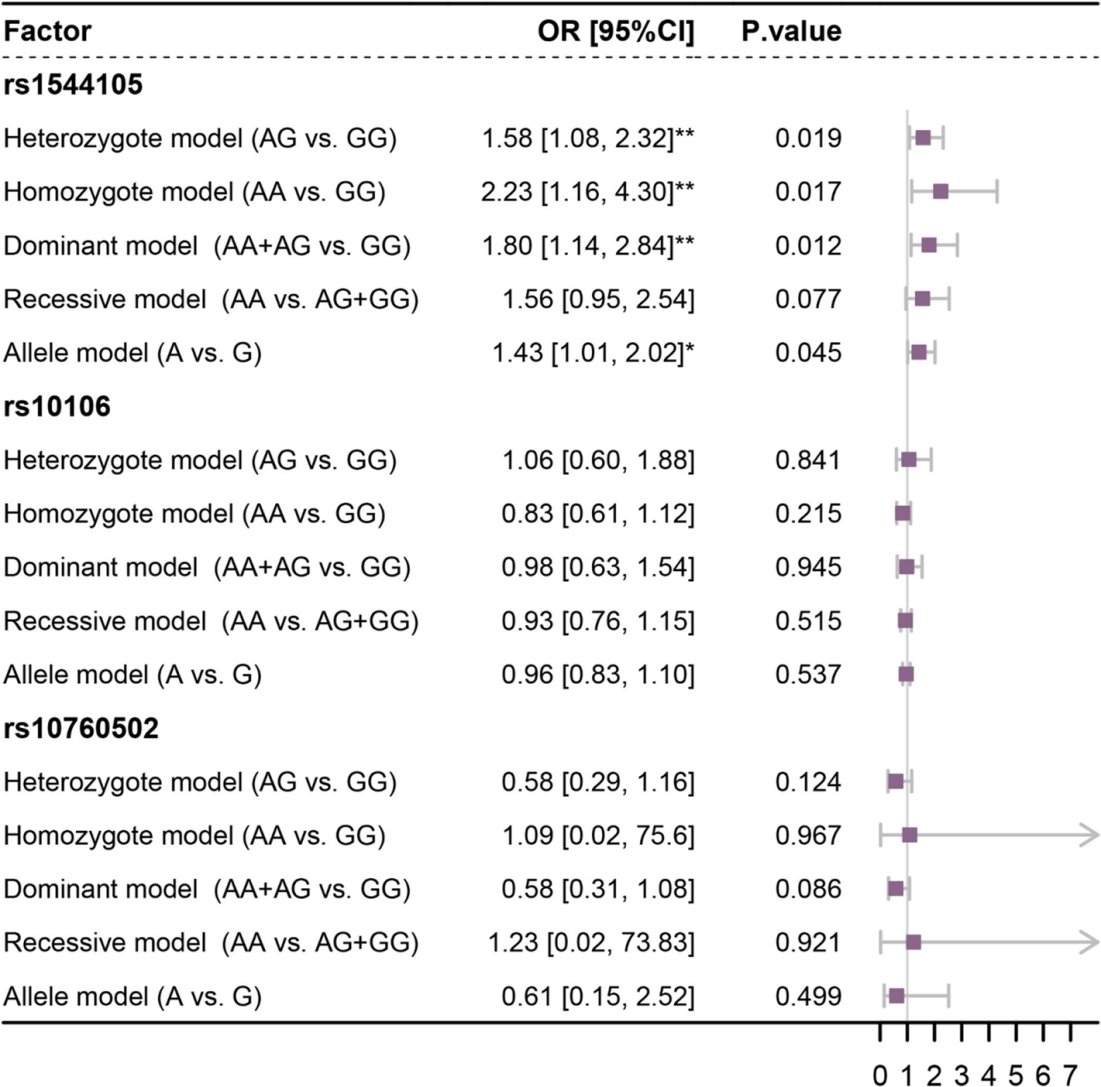
Forest plot of the association between FPGS SNP sites polymorphism and ALL disease progression. This forest plot illustrates the pooled ORs and 95% CIs for the association between FPGS SNPs and ALL progression. Each included study is indicated by a square, with the horizontal line representing its 95% CI. The size of the square reflects the relative weight of the study in the meta-analysis. The diamond represents the pooled OR with its 95% CI. A positive OR value indicates an increased risk of disease progression, while a negative OR value indicates a decreased risk of disease progression

Seven studies [26, 30–35], involving three SNP sites (rs1544105, rs10106, rs10760502) were included in the meta-analysis of ALL progression. The heterogeneity analysis indicated that significant homogeneity (I^2^ < 50% and *p* > 0.1) was observed only in the homozygote model (AA vs. GG), recessive model (AA vs. AG + GG), and allele model (A vs. G) of rs10106, as well as the heterozygote model (AG vs. GG) of rs10760502 (Supplementary Table 3). Therefore, a common-effect model was applied to these comparisons, while a random-effect model was used for all other analyses. In the allele model, compared with the G allele, the rs1544105 A allele was associated with a 1.43-fold increased risk of ALL progression (95% confidence interval [CI]: 1.01–2.02; *p* = 0.045). In other models, when the rs1544105 locus contained the A allele (AG, AA, or AG + AA), the incidence of ALL progression was at least 1.5 times (odds ratio [OR]) greater than that for the homozygous GG genotype (*p* < 0.05). Specifically, the disease progression rate for the homozygous AA genotype was significantly greater (OR = 2.23) than that for the homozygous GG genotype (95% CI: 1.16–4.30; *p* = 0.017). Other SNPs (rs10106, rs10760502) showed no significant association (Fig. 2)*.*

**Fig. 3:**
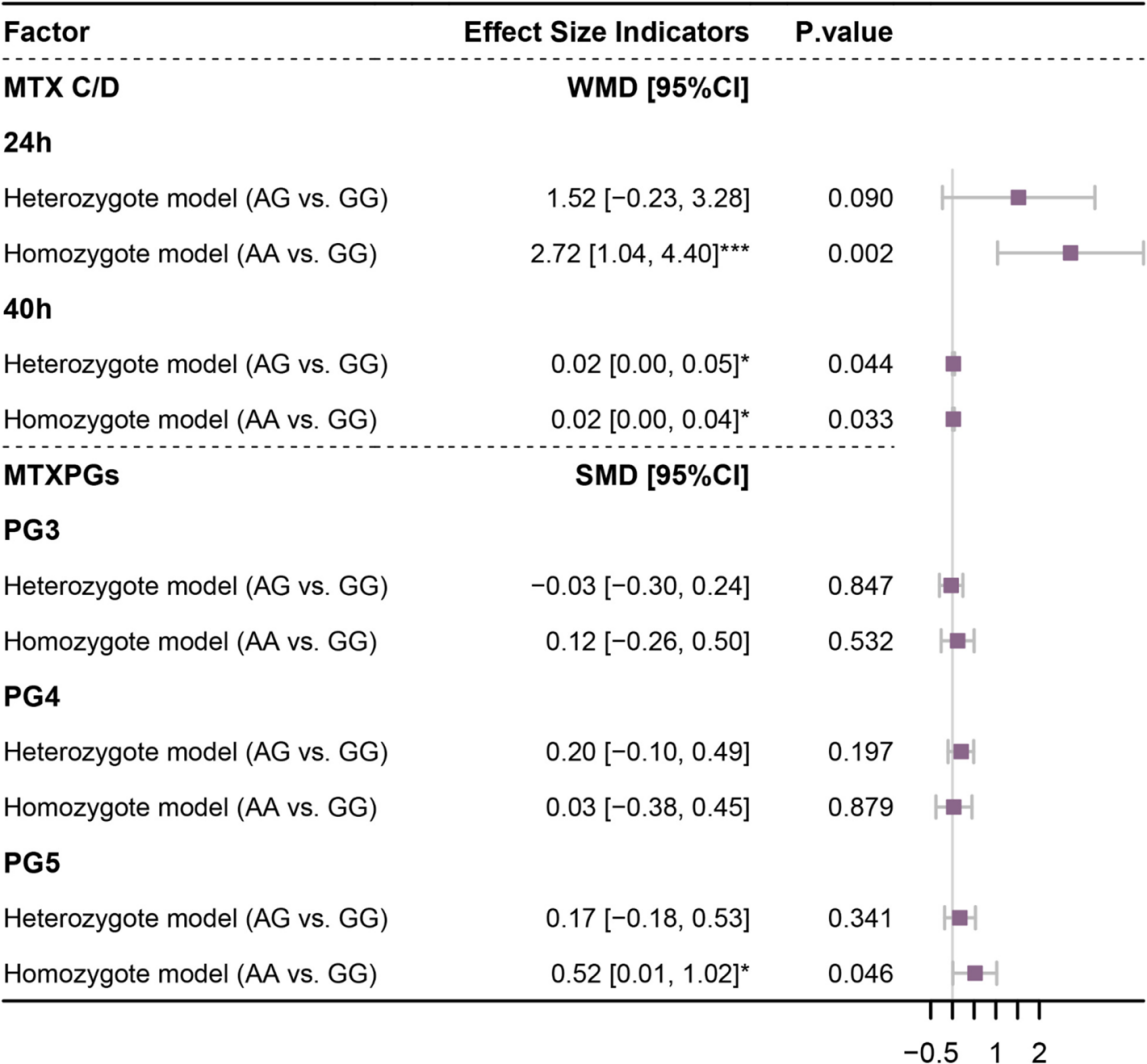
Forest plot of the association between FPGS rs1544105 polymorphism and MTX efficacy index. This forest plot illustrates the pooled WMD and SMD and 95% CIs for the association between FPGS rs1544105 and MTX efficacy index. Each study is represented by a square, with the size proportional to its weight in the meta-analysis, while the horizontal lines indicate the corresponding 95% CIs

A total of four studies [28, 36–38] were included in the meta-analysis of the association between FPGS rs1544105 polymorphisms and MTX efficacy. On the basis of the data type, the correlation between the FPGS rs1544105 polymorphism and MTX concentration-to-dose (C/D) ratio at 24 and 40 h post-administration was analyzed using the weighted mean difference (WMD) effect size, whereas the correlation between FPGS rs1544105 polymorphism and methotrexate polyglutamates 3–5 (MTXPG3–5) concentrations post-administration was analyzed using the standardized mean difference (SMD) effect size. The heterogeneity analysis showed that all comparisons demonstrated homogeneity (I^2^ < 50% and *p* > 0.1, Supplementary Table 4), and therefore a common-effect model was applied to all analyses. The results showed that in the homozygous model, when the rs1544105 locus was the AA genotype, the MTX C/D ratios at 24 (2.72, 95% CI: 1.04–4.40, *p* = 0.002) and 40 h (0.02, 95% CI: 0.00–0.04, *p* = 0.033) after MTX administration were significantly greater than those with the GG genotype. When the rs1544105 locus was the AG genotype, MTX levels at 40 h (0.02, 95% CI: 0.00–0.05, *p* = 0.044) were significantly higher compared to the GG genotype, and although no significant difference was observed at 24 h (1.52, 95% CI: −0.23–3.28, *p* = 0.090), a slight increase was still noted. However, the MTXPG levels did not significantly differ between the two models, except for MTXPG5 (*p* > 0.05), indicating that the homozygous GG genotype is more favorable for MTX absorption (Fig. 3).

A funnel plot was used to investigate the potential impact of publication bias on the results (Fig. S1, S2). Asymmetry in the funnel plot suggests the possibility of publication bias or the influence of smaller studies with relatively greater variance in the research on the association between the FPGS rs1544105 polymorphism and ALL disease progression (Fig. S1a). However, our studies were predominantly concentrated in the middle section of the funnel plot, indicating a positive aspect of data reliability. Sensitivity analysis was performed by sequentially omitting individual studies to reassess the impact of each study on the pooled OR, WMD, or SMD (Fig. S3, S4). The results indicated that omitting any single study did not cause significant differences, confirming the stability and reliability of the findings in this study.

### rs1544105 as an expression quantitative trait locus (eQTL) of the FPGS gene

To determine whether rs1544105 functions as an eQTL for FPGS, we analyzed this site using three online eQTL databases that integrate diverse human datasets. The results consistently demonstrated that rs1544105 is a cis-eQTL of FPGS (*p* = 8.7 × 10⁻^16^ to 1.1 × 10⁻^10^, False discovery rate [FDR] < 0.05, Supplementary Table 5). Based on these findings, subsequent experimental validation focused on the impact of rs1544105 polymorphism on FPGS gene expression.

### Constructed mutation-293T (Mut-293T) cells exhibit significant upregulation of FPGS mRNA and protein

**Fig. 4:**
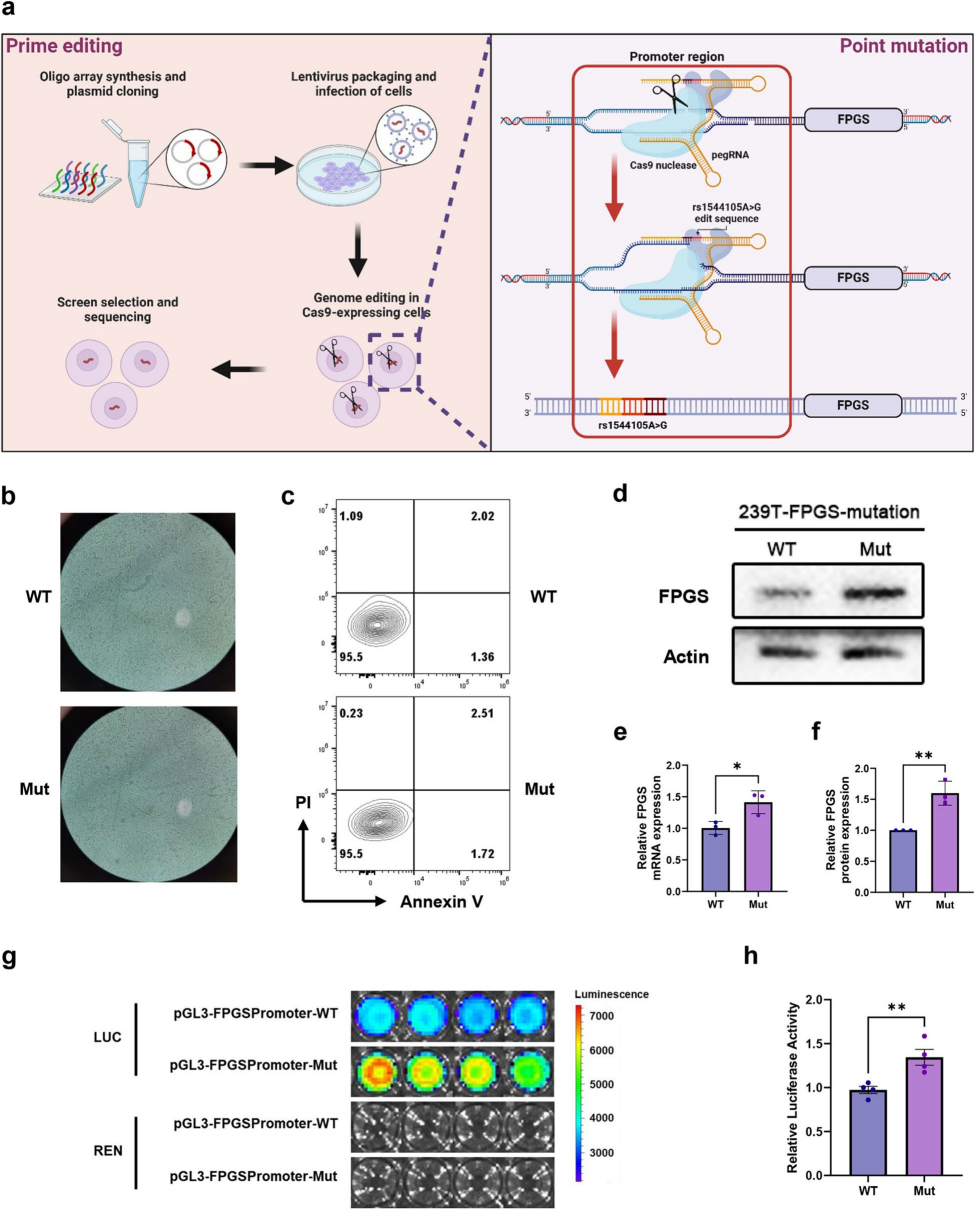
Construction of FPGS rs1544105 A > G 293T cell gene editing models. **a** Schematic representation of the PE strategy used to generate FPGS rs1544105 A > G mutations in 293T cells. **b**, **c** Microscopic and flow cytometry images comparing the morphology and fluorescence signals of WT-293T and Mut-293T cells. **d** WB analysis showing FPGS protein expression levels in WT-293T and Mut-293T cells. **e**, **f** Quantitative comparison of FPGS mRNA and protein expression levels between WT-293T and Mut-293T cells (*n* = 3). **g** In vivo imaging of cells transfected with dual-luciferase reporter plasmids. **h** Relative luminescence intensity of cells in the dual-luciferase reporter assay (*n* = 4). Statistical significance was determined using a two-tailed Student’s t-test (*p < 0.05, **p < 0.01)

To generate a cell line carrying the rs1544105 mutation, we first performed PCR on the FPGS rs1544105 locus in wild-type 293T cells (WT-293T) using specific primers. The result showed that WT-293T cells exhibited a homozygous AA genotype at rs1544105 (Fig. S5). Consequently, the TGG sequence, located 12 bp downstream of the rs1544105 site, was selected as the PAM sequence. Based on this finding, two epegRNA plasmids, termed FPGS_19_AtoG_q1/not, were constructed using two different 3' motifs, tevopreq1 and tmpknot. 293T cells were subsequently transfected with PEmax-GFP and the FPGS_19_AtoG_q1/not plasmids. Following fluorescence-activated cell sorting (FACS) and single-clone screening, the FPGS rs1544105 homozygous GG (rs1544105 A > G) 293T cell line (Mut-293T) was successfully established (Fig. 4a). Upon continuous passaging, Mut-293T cell maintained a stable morphology and viability. (Fig. 4b, c). In addition, STR profiling of WT-293T cells demonstrated a match with the HEK293T cell line recorded in the EXPASY database, with an EV value of 0.94 (Supplementary Table 6). Mycoplasma testing confirmed that both WT-293T and Mut-293T cells were free of contamination during culture (Fig. S6).

RT-PCR and quantitative real-time PCR (qPCR) and Western blot (WB) showed ~ 1.5-fold upregulation of FPGS mRNA and protein in Mut-293T compared with WT-293T (*p* < 0.05, Fig. 4d-f), while neighboring genes were unaffected (Fig. S7). This finding provides the cell-level evidence that, when compared with the homozygous AA genotype, the homozygous GG genotype at the rs1544105 locus is associated with significantly higher FPGS expression.

### Mut-293T cells increase FPGS expression by enhancing transcriptional activity via modulation of transcription factor binding

To explore the molecular mechanism underlying the elevated FPGS expression in Mut-293T cells compared with WT-293T cells, we used the MoLoTool and identified that the sequence harboring rs1544105 (TTCGACGTAAC) has the potential to bind the transcription factor cAMP responsive element binding protein 1 (CREB1, *p* = 5.7 × 10⁻^3^; Fig. S8). WB confirmed similar nuclear CREB1 levels in both cell lines (Fig. S9). Electrophoretic Mobility Shift Assay (EMSA) with recombinant CREB1 showed stronger binding to the rs1544105 G probe than to the wild-type probe, whereas binding to the wild-type probe was negligible (Fig. S10). These findings indicate that the rs1544105 mutation may regulate FPGS expression by altering the binding affinity of this region for CREB1. It is noteworthy that CREB1 antibody–supershift bands were not detected in the EMSA, likely due to competitive binding between the probe and the antibody for recombinant CREB1 under non-denaturing gel conditions, which prevented the antibody from binding to the probe-bound CREB1 protein (Fig. S9, S10).

To directly assess whether the GG genotype of rs1544105 enhances transcriptional activity compared with the AA genotype, we performed a dual-luciferase reporter assay and imaged the transfected cells. The results showed that cells carrying the rs1544105 G exhibited significantly stronger bioluminescent signals than those carrying the rs1544105 A (Fig. 4g). After normalization to the internal Renilla luciferase control, the relative bioluminescent signal of the rs1544105 Mut genotype remained significantly higher than that of the WT genotype (Fig. 4h). These results indicate that the GG genotype of rs1544105 can enhance FPGS expression by increasing transcriptional activity relative to the AA genotype.

### Mut-293T cells significantly enhance the in vitro efficacy of MTX

**Fig. 5:**
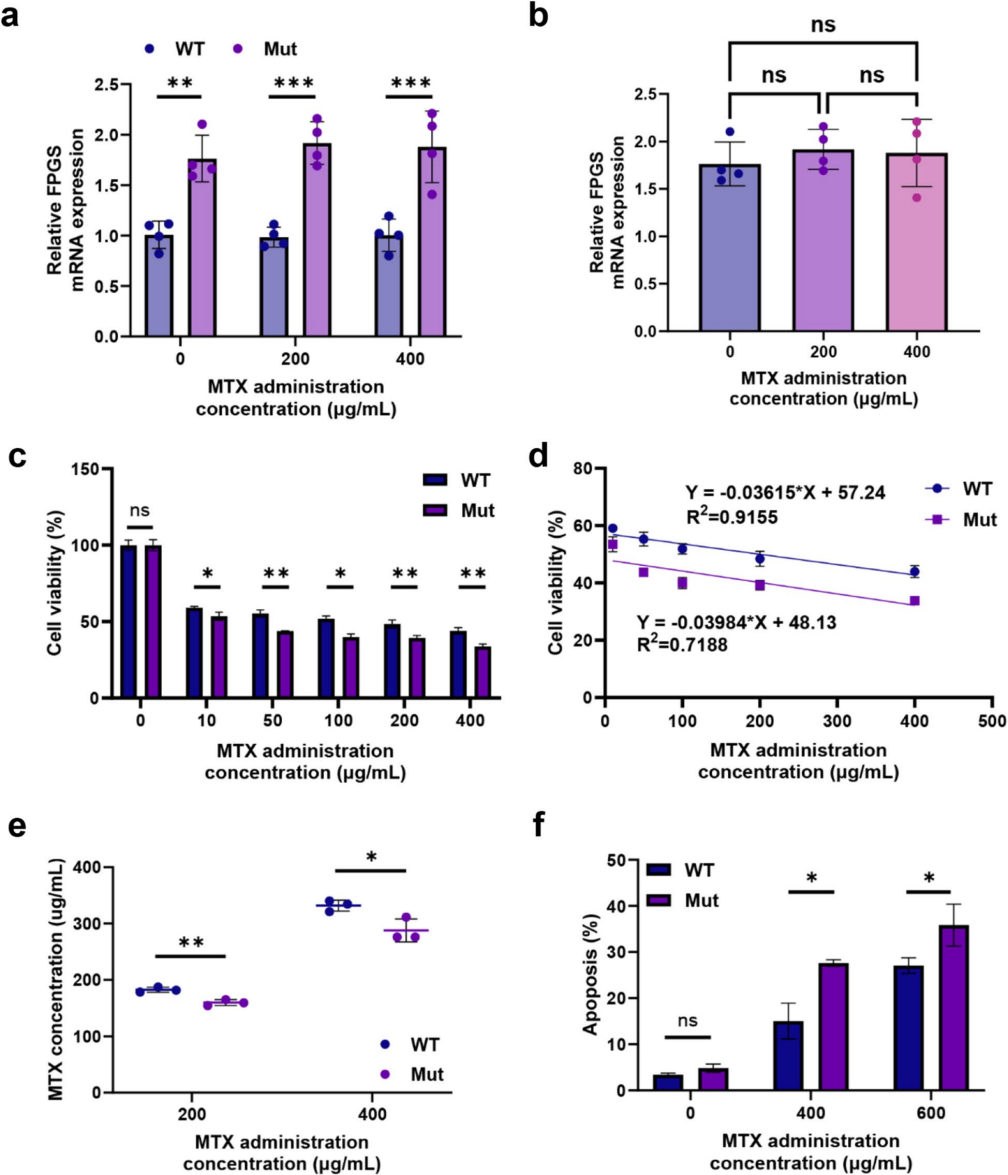
Differences in MTX efficacy between WT-293T and Mut-293T cells in vitro. **a**, **b** FPGS gene expression levels in WT-293T and Mut-293T cells under different concentrations of MTX treatment (*n* = 4). **c**, **d** Cell viability of WT-293T and Mut-293T cells under varying concentrations of MTX treatment (*n* = 3). **e** Extracellular MTX concentrations in WT-293T and Mut-293T cells under different MTX treatment conditions (*n* = 3). **f** Apoptotic cell proportions in WT-293T and Mut-293T cells under different MTX treatment conditions (*n* = 3). Statistical significance was determined using a two-tailed Student’s t-test and Simple Linear Regression (*p < 0.05, **p < 0.01, ***p < 0.005, ns = not significant)

To evaluate whether the FPGS gene in WT-293T and Mut-293T cells is influenced or regulated by MTX drug pressure, the two cell lines were cocultured with 0–400 µg/mL MTX. The results revealed that FPGS gene expression level in both cell lines was not affected by the MTX concentration (Fig. 5a, b).

To assess the impact of the FPGS gene rs1544105 GG and AA genotypes on MTX efficacy, MTX efficacy experiments were conducted on WT-293T and Mut-293T cells. Cell counting kit‑8 (CCK8) assay revealed that compared with WT-293T cells, Mut-293T cells had significantly lower cell viability (Fig. 5c), and the cell viability of Mut-293T cells decreased more rapidly at the same MTX concentration (Fig. 5d). Extracellular MTX accumulation was higher in Mut-293T cultures (Fig. 5e) (Fig. S11), and apoptosis assays confirmed higher apoptotic ratios under MTX treatment (Fig. 5f). These findings indicated that the Mut-293T cells are more sensitive to MTX.

### Mut-293T cells significantly enhance MTX efficacy in xenograft models

**Fig. 6:**
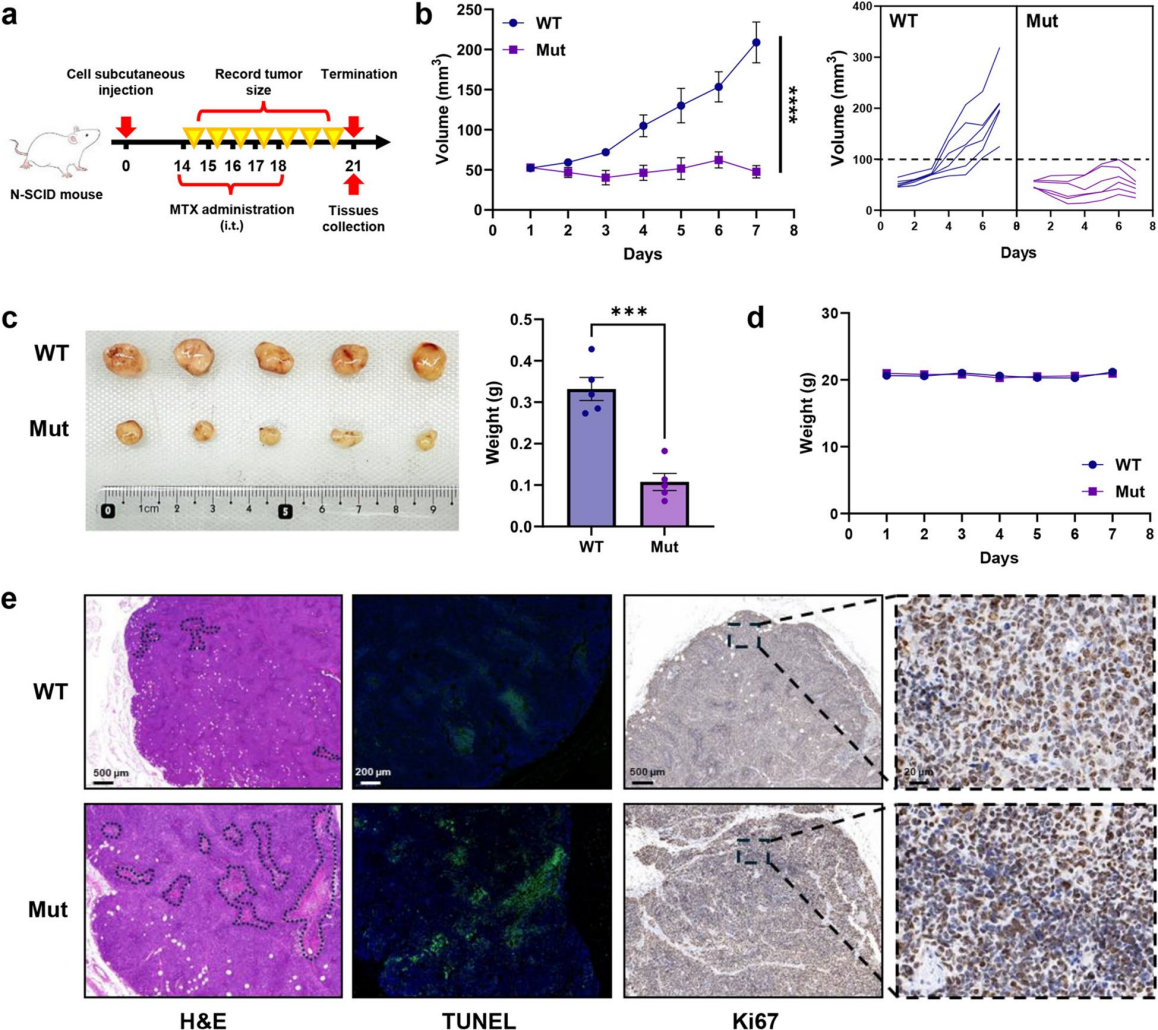
Differences in MTX efficacy between WT-293T and Mut-293T cells in vivo. **a** Schematic timeline of the experiment to evaluate MTX efficacy in animal models based on WT-293T and Mut-293T cells. **b** Tumor volume growth curves (*n* = 5). **c** Photographs of tumors at the experimental endpoint and corresponding tumor weight differences (*n* = 5). **d** Body weight changes of mice during the experiment (*n* = 5). **e** Histological analyses of tumor tissues including H&E staining, TUNEL fluorescence staining, and Ki67 immunohistochemistry. Statistical significance was determined using a two-tailed Student’s t-test and Simple Linear Regression (***p < 0.005, ****p < 0.001)

To investigate the in vivo antitumor efficacy of MTX, xenograft models were generated by injecting WT-293T and Mut-293T cells into N-SCID mice (Fig. 6a). After one week of MTX treatment, tumor growth in the Mut-293T xenograft model was significantly slower than that in the WT-293T model (*p* < 0.001, Fig. 6b). On day 7 post-treatment, the tumor weight in the Mut-293T group was only 0.3-fold of that in the WT-293T group (*p* < 0.005, Fig. 6c), while no significant differences in body weight were observed between the two groups (*p* > 0.05, Fig. 6d). Histological analyses confirmed larger necrotic areas, higher apoptosis, and reduced tumor cell viability in Mut-293 T-derived tumors (Fig. 6e).

**Fig. 7:**
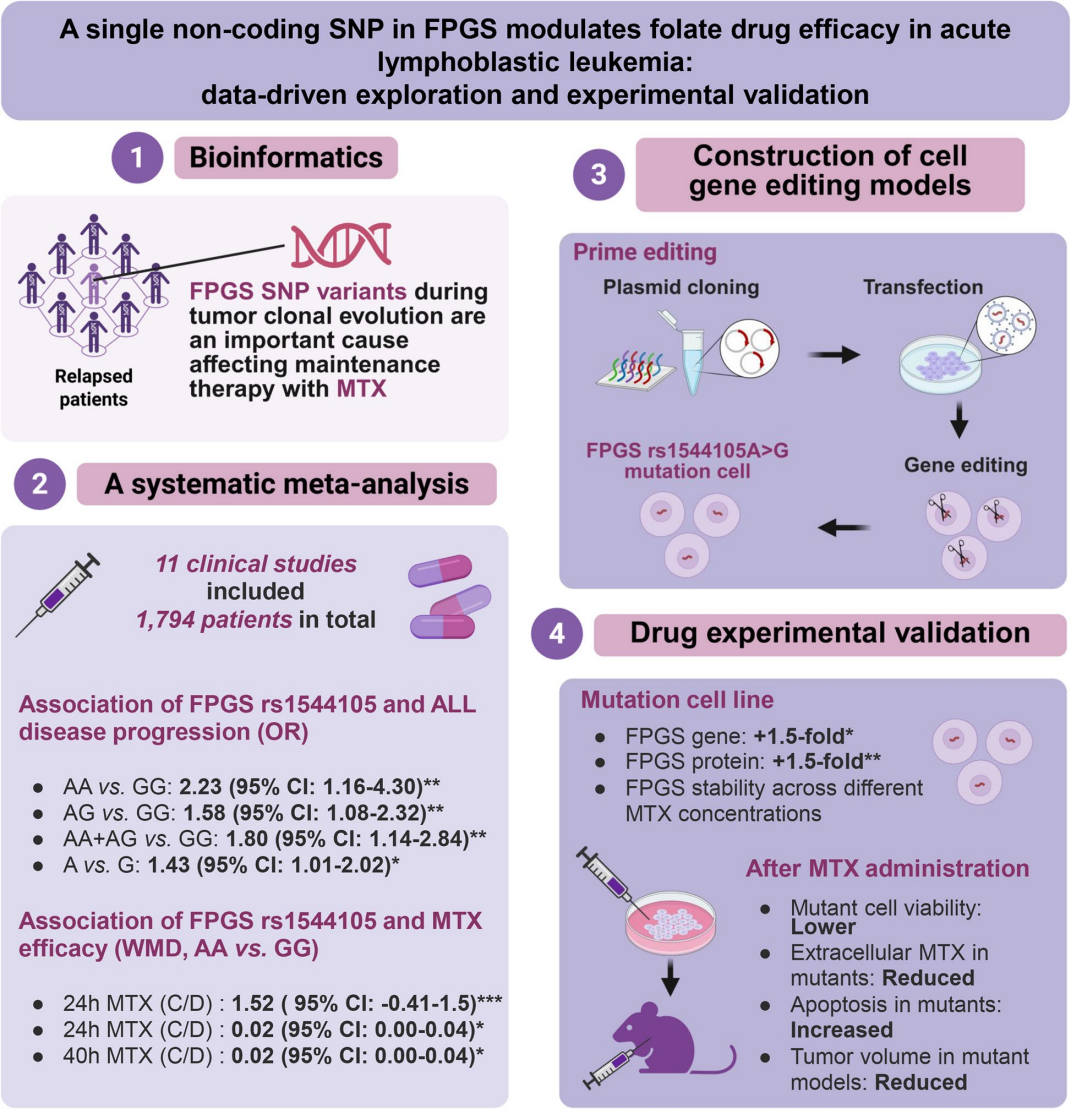
Schematic illustration of the study design and main findings. The figure illustrates the overall experimental design and major findings regarding the effects of FPGS rs1544105 polymorphisms on MTX-related clinical indices and therapeutic efficacy

**Fig. 8:**
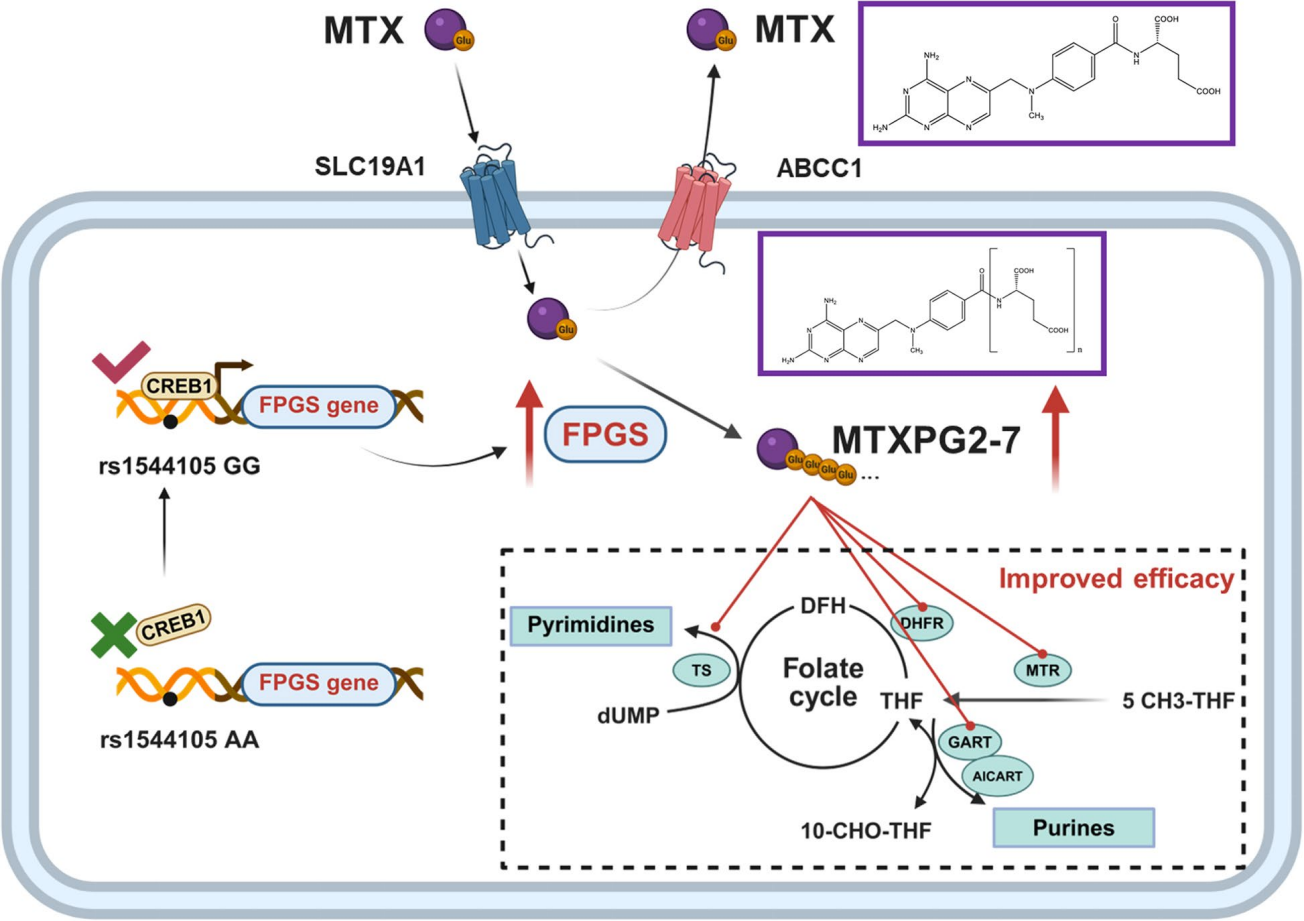
Mechanistic illustration. The rs1544105 locus changing from the AA genotype to the GG genotype increases the binding affinity of the transcription factor CREB1 to this region, thereby enhancing the transcriptional efficiency of the FPGS gene and increasing FPGS protein expression. Elevated FPGS protein levels further promote the conversion of MTX to MTXPG2–7 and enhance MTX intracellular retention, ultimately leading to improved antitumor efficacy

This study first bioinformatically elucidated the potential significant impact of mutations at the FPGS gene locus on poor prognosis in ALL patients receiving MTX maintenance therapy. The subsequent meta-analysis focused on the rs1544105 locus in the FPGS gene promoter, revealing that ALL patients with the homozygous AA genotype at this site had a higher rate of disease progression and poorer MTX-related efficacy than patients with the GG genotype. Then a CRISPR-based gene editing approach was used to construct a Mut-293T cell with the GG genotype at rs1544105 from WT-293T cells with the AA genotype. Through experimental validation including cellular experiments, EMSA, dual-luciferase reporter assays, and animal studies, we demonstrated that the GG genotype at rs1544105 positively regulates FPGS transcription by enhancing transcription factor binding, thereby increasing FPGS mRNA and protein levels (Fig. 7). This upregulation augments intracellular FPGS catalytic function, enhances MTX efficacy at the same dose (Fig. 8), and ultimately ensures the effectiveness of MTX maintenance therapy.

## Discussion

Although advancements in pharmacogenomics have led to the identification of numerous chemotherapy resistance-related genes and SNP loci, the integration of genetic polymorphism research with traditional chemotherapy in clinical practice remains limited [39, 40]. Many genetic polymorphisms or mutations are identified solely through association studies without further refinement or experimental validation, limiting their clinical utility in guiding personalized drug regimens and improving patient outcomes. This study explores the impact of FPGS-related SNPs on MTX efficacy in ALL patients. Through an integrated approach combining bioinformatics, systematic meta-analysis, gene editing, in vitro and in vivo pharmacodynamic experiments, and mechanistic investigations, we provide multidimensional evidence on the functional impact of the rs1544105 locus in the FPGS non-coding region. The GG genotype at this site enhances the binding affinity of the transcription factor CREB1, thereby increasing transcriptional activity. As a result, FPGS gene and protein expression are significantly elevated, leading to enhanced intracellular MTX retention. Ultimately, these effects contribute to better MTX efficacy compared with the AA genotype. Thus, the AA genotype at rs1544105 may be a key determinant of suboptimal MTX maintenance therapy outcomes in ALL patients.

Previous studies have demonstrated that MTX treatment exerts strong selective pressure on folate polyglutamylation in ALL cells [25], driving mutations in the FPGS gene (Fig. 1a) [41]. This process promotes MTX resistance by downregulating FPGS expression or rendering it inactive, ultimately contributing to relapse. However, since FPGS is likely an essential gene in many eukaryotic cells [42], tumor cells must strike a balance, reducing MTX activity without compromising their own survival. As a result, complete inactivation or loss of FPGS function is not a viable strategy. The rs1544105 polymorphism in the FPGS promoter region, identified through our systematic meta-analysis, precisely aligns with this scenario. Under the strong selective pressure of MTX treatment, tumor cells can attenuate FPGS expression by approximately 1.5-fold with only a "minimal mutational cost"—a single SNP alteration—thereby weakening MTX efficacy without completely compromising their own survival. However, based on current studies [26–28], rs1544105 is a typical germline SNP site, and it remains unclear whether MTX administration pressure can directly induce it to become a somatic mutation. Therefore, the clinical significance of our findings mainly lies in providing pharmacogenomic guidance for MTX maintenance therapy in ALL patients—specifically, MTX is not recommended as a maintenance drug for patients carrying the homozygous AA genotype at this locus. Notably, given that the gnomAD v4.1.0 database reports a high allele frequency of 48.3% for rs1544105 G > A among 151,998 alleles [29], our finding reveal a common pharmacogenomic mechanism with broad applicability across ALL patients.

Our systematic meta-analysis of the rs1544105 polymorphism revealed that compared with the GG genotype, the presence of the A allele, whether in the homozygous or heterozygous state, is associated with an increased risk of ALL disease progression and a significant increase in MTX C/D ratio. This finding is consistent with several previous association studies [26, 27]; however, our study is the first to experimentally confirm, through both cellular and animal models, that the AA genotype indeed reduces MTX efficacy. Furthermore, we identified that this effect likely results from weakened binding between the CREB1 transcription factor and the FPGS promoter region containing the rs1544105 locus, thereby decreasing transcriptional efficiency of the FPGS gene. The elucidation of this regulatory mechanism provides a potential therapeutic approach: interventions could be developed to enhance FPGS transcription in patients with the AA genotype, thereby improving MTX efficacy.

Fortunately, in ALL patients with inherently low FPGS expression and those exhibiting reduced FPGS levels due to mutation, the consequent impairment in folate metabolism may serve as an Achilles' heel for ALL cells. Previous studies have demonstrated that lipophilic antifolates such as trimetrexate, which do not rely on polyglutamation for activation, exhibit potent efficacy against ALL cells with diminished FPGS expression, suggesting a promising therapeutic alternative for this subset of patients [25].

From a methodological perspective, traditional approaches for validating SNP biological significance typically involve generating knockout cell lines for SNP-associated proteins, followed by transfection with expression plasmids carrying the mutant sequence to restore protein expression. Functional assays are then performed using these modified cells [43]. However, this classical approach has inherent limitations, not only because of potential off-target mutations introduced during the knockout and complementation processes, but also because it is restricted to validating point mutations located within exonic regions, leaving mutations in noncoding genomic regions unaddressed. In this study, the identified rs1544105 site is located within the non-coding region, more than 2.4 kb upstream of the FPGS start codon, making it impossible to generate a suitable cellular model using conventional methods. To overcome this challenge, we employed PE to directly convert the AA genotype to GG at the rs1544105 locus in the genomic DNA of WT-293T cells, establishing the Mut-293T cell line for subsequent functional validation. This approach not only provides the first direct evidence that the rs1544105 polymorphism or mutation modulates MTX efficacy in ALL by regulating FPGS gene and protein expression levels but also establishes a robust foundation and experimental platform for future pharmacogenomic studies targeting non-coding single-nucleotide variants.

Despite our comprehensive research approach, this study has certain limitations. For example, the sample size in our bioinformatics analysis was relatively small, which prevented us from conducting subgroup analyses by ethnicity, although ethnic differences are a critical factor influencing the outcomes of SNP association studies; in the systematic meta-analysis, we were unable to determine whether disease progression or occurrence in ALL patients is directly associated with MTX resistance, and owing the low transfection efficiency of PE plasmids in human-derived ALL cells, we ultimately used a non-ALL human 293T cell line to construct the mutant cell model and animal model. Nevertheless, we believe that future large-scale and more refined big data studies in ALL populations will further substantiate our current findings, and that additional experiments in human-derived ALL cells will provide further validation of our conclusions.

In summary, this study focuses on the critical challenge of pediatric ALL clinical treatment relapse caused by poor treatment efficacy or resistance to MTX maintenance therapy. By integrating multiple research approaches, including bioinformatics, systematic meta-analysis, gene editor, molecular and cellular biology experiments, and animal studies, we investigated the polymorphism of the rs1544105 locus in MTX efficacy-related genes. Our results indicate that, compared with the GG genotype, the AA genotype at rs1544105 significantly reduces the binding of the surrounding sequence to the transcription factor CREB1, thereby lowering transcriptional efficiency and decreasing FPGS gene and protein expression. This limits MTX efficacy. These findings provide valuable insights for advancing precision medicine in ALL and overcoming the challenge of relapse, with important clinical implications.

## Materials and methods

### Bioinformatics analysis

Previously published serial deep sequencing data of patient ALL028 were reanalyzed to infer clonal evolution patterns [25, 41, 44], based on the time of appearance and mutation allele frequency. The analysis specifically focused on identifying FPGS mutations and associated genetic alterations, which were visualized using the “fishplot” package in R software 4.4.3 (R Foundation, Vienna, Austria).

### Systematic meta-analysis

This systematic meta-analysis was conducted in accordance with the PRISMA guidelines (Supplementary Table 7) [45, 46] and was prospectively registered in PROSPERO (CRD42024501323). Inclusion criteria were formulated following the PICO model (Population, Intervention, Comparison, and Outcomes). The methodological quality of eligible studies was assessed using the NOS scale [47].

#### Search strategy

We conducted a systematic search of Embase, PubMed, Medline, Wanfang, and CNKI databases up to February 2, 2025, using Medical Subject Headings terms including “leukemia,” “acute lymphoblastic leukemia,” “mutation,” “variant,” “polymorphism,” “folate,” “FPGS,” and “folylpolyglutamate synthase,” without language restriction. Detailed search strategies for each database are provided in Supplementary Table 8. Additionally, the reference lists of the primary studies were reviewed by two authors (WY and YL).

#### Study selection

In accordance with the PRISMA protocol, two reviewers (WY and YL) independently conducted the search and screened the identified literature for relevance based on the predefined inclusion and exclusion criteria, utilizing EndNote 21 [48].

Studies that included both FPGS gene-related SNP sequencing information and data on ALL disease progression or MTX-related pharmacodynamic indicators were selected based on the title and abstract. The specific exclusion criteria were as follows: 1) duplicate publications; 2) nonclinical studies; 3) lack of SNP data; 4) theses, editorials, conference abstracts, protocols, case reports, or reviews; 5) genotype frequencies in controls not adhering to HWE; and 6) incomplete or invalid datasets [49].

#### Initial review

The initial database was compiled, and all duplicate articles were removed. Citations were screened based on the title, abstract, and the previously defined inclusion criteria. Only after a full-text assessment by two authors (WY and YL) were the studies ultimately selected for inclusion in the review. Any disagreements were resolved through discussion [49].

#### Data extraction

The data for the initial review were recorded independently by both authors using a standard data extraction form. The following information was collected from each study: the name of the first author, publication year, ethnicity, patient age, MTX dose, control source, genotyping methods, number of cases and controls, genotype distribution, HWE *P* value, MTX C/D ratio, and MTXPGs concentration.

#### Quality assessment

Two investigators independently evaluated study quality using the NOS scoring system. Only studies with NOS scores > 5 were included [47]. Discrepancies were resolved through discussion.

#### Statistical analysis

The *P*-value for HWE among the control groups for each study was calculated using the Chi-square test. Pooled ORs and 95% CIs were estimated under five genetic comparison models: the heterozygote model (AG vs. GG), homozygote model (AA vs. GG), dominant model (AA + AG vs. GG), recessive model (AA vs. AG + GG), and allele model (A vs. G). These models were used to assess the association between FPGS polymorphisms and ALL disease progression. The heterozygote and homozygote models were specifically employed to evaluate the association of FPGS polymorphisms with MTX-related efficacy indices.

Pooled ORs, SMDs, and 95% CIs were used to evaluate the impact of FPGS polymorphisms on ALL disease progression and MTX-related efficacy indices, respectively. WMD was used for data with the same measurement units, while SMD was used for data with different measurement units and large differences in means. The formulas are as follows:

$$\text{WMD}= {\overline{\text{x}} }_{\text{e}}-{\overline{\text{x}} }_{\text{c}}$$

$$\text{SMD}=\frac{{\overline{\text{x}} }_{\text{e}}-{\overline{\text{x}} }_{\text{c}}}{{\text{s}}_{\text{within}}}$$

where: ${\overline{\text{x}} }_{\text{e}}$ is the sample mean of the case group (e.g., AAcase or AGcase), ${\overline{\text{x}} }_{\text{c}}$ is the sample mean of the control group (e.g., GGcon), ${\text{s}}_{\text{within}}$ is the pooled within-group standard deviation across groups:

$${\text{s}}_{\text{within}}=\sqrt{\frac{\left({\text{n}}_{\text{e}}-1\right){\text{s}}_{\text{e}}^{2}+({\text{n}}_{\text{c}}-1){\text{s}}_{\text{c}}^{2}}{{\text{n}}_{\text{e}}+{\text{n}}_{\text{c}}-2}}$$

where: ${\text{s}}_{\text{e}}$ is the standard deviation of the case group, ${\text{s}}_{\text{c}}$ is the standard deviation of the control group, ${\text{n}}_{\text{e}}$ and ${\text{n}}_{\text{c}}$ are the numbers of patients in the case and control groups, respectively.

Pooled SMDs were computed and weighted for the sample size of the individual studies. These pooled effect sizes were classified as small (0.2), moderate (0.5), and large (0.8) [50, 51].

The Z-test was applied to compare the effects between the case and control groups. If the *P*-value was less than 0.05, the pooled ORs and SMDs were considered statistically significant. Subsequently, the Q test and I^2^ statistics were used to assess the heterogeneity of the included studies. A *P*-value ≤ 0.1 and/or I^2^ ≥ 50% indicated significant heterogeneity, and the random-effects model was used for data calculation. A *P*-value > 0.1 and/or I^2^ < 50% indicated no significant heterogeneity, and the common-effects model was applied for data calculation [52].

For studies reporting medians and ranges, mean ± SD values were estimated using an online converter (https://www.math.hkbu.edu.hk/~tongt/papers/median2mean.html) [53–55]. Publication bias was evaluated via funnel plots, Begg’s and Egger’s tests, and sensitivity analysis was performed by stepwise omission of individual studies.

### eQTL query

We queried the rs1544105 locus using three online eQTL analysis platforms (eQTLGen Consortium, GTEx Portal, and eQTL Catalogue) and collected information on dataset sources, associated genes, *P* values, and FDR values. A locus was defined as an eQTL for the corresponding gene when the *p* value was less than 1 × 10⁻^5^ and the FDR was below 0.05 [56–58].

### Construction of mutation cell

#### Plasmid construction

The plasmids used in this study were purchased from Addgene (Watertown, MA, USA), including: PEmax-GFP plasmid (pCMV-PEmax-P2A-GFP, #180020), and two empty plasmids for epegRNA, namely pU6-tevopreq1-GG-acceptor (#174038) and pU6-tmpknot-GG-acceptor (#174039).

To construct the epegRNA expression plasmids, four pairs of oligonucleotides were designed and ordered from GenScript Biotech Co., Ltd. (Nanjing, Jiangsu, China): FPGS_Spacer, an improved scaffold [59], and two 3' extension sequences (FPGS_19AtoG_q1 and FPGS_19AtoG_not). The sequences are listed in Supplementary Table 9, 10. These oligonucleotides were annealed to form four double-stranded DNA fragments with sticky ends. The T4 PNK kinase (Thermo, #EK0031) phosphorylated the scaffold ends, and BsaI (Thermo, #FD0294) was used to digest the empty epegRNA plasmids. The digested plasmids, FPGS_Spacer, scaffold, and the 3' extension sequences were ligated using T4 ligase (Thermo, #EL0014). Ligation products were transformed into DH5α cells, plated on LB agar with Ampicillin, and incubated overnight at 37 °C. Single colonies were verified by colony PCR using epegRNA_F/R primers and analyzed by agarose gel electrophoresis. The correctly sized PCR products (~ 560 bp) were sequenced by Nanjing Qingke Biotech Co., Ltd. (Nanjing, Jiangsu, China), and the validated recombinant plasmids were named FPGS_19_AtoG_q1 and FPGS_19_AtoG_not.

#### Cell culture and transfection

The HEK293T (ATCC CRL-3216, Shanghai Cellsolution Biotech Co., Ltd., Shanghai, China) were cultured in Dulbecco’s Modified Eagle’s Medium with 10% (v/v) fetal bovine serum (Gibco, qualified), 1 × penicillin–streptomycin and glutamine (Thermo, #35050061) mixture (DMEM +/+) at 37 °C with 5% CO_2_. Cell line authentication was performed by Yimo Biotechnology Co., Ltd. (Nanjing, Jiangsu, China) using STR profiling. A panel of 23 STR loci, including Amelogenin and Yindel, was analyzed to verify the genetic identity and quality of the cell lines.

WT-293T cells were seeded in 48-well plates and cultured for 20 h. Then, 1.5 mL antibiotic-free DMEM (DMEM +/-) was added to each well. A mixture of 750 ng PEmax-GFP and 250 ng FPGS_19_AtoG_q1/not was combined with 250 µL Opti-MEM (Thermo, #31985070) and incubated at room temperature for 5 min. 2 µL of Hieff Trans Liposomal Transfection Reagent (YEASEN, #40802ES03) was added to another 250 µL Opti-MEM and incubated for 5 min. The two solutions were mixed and incubated for 20 min before adding to each well. After 8 h, the medium was replaced with fresh complete DMEM (DMEM +/+). WT-293T as control was transfected only PEmax-GFP without FPGS_19_AtoG_q1/not. Post-passage, FACS was used to sort EGFP-positive cells. In this study, Mut-293T is a monoclonal cell line of cells transfected with FPGS_19_AtoG q1 plasmid.

#### DNA extraction and sequencing

Cells (2 × 10^5^) were washed with PBS and lysed with 500 µL SNET buffer (1% SDS, 400 mM NaCl, 5 mM EDTA, 20 mM Tris–Cl, pH 8.0) and 1 µL proteinase K at 56 °C for 8 h. After cooling, the lysate was mixed with 1 mL phenol/chloroform and centrifuged at 12,000 rpm for 10 min. The supernatant was collected and DNA precipitated with 3 mL ethanol, followed by centrifugation. The DNA pellet was washed with 70% ethanol, air-dried, and dissolved in 100 µL TE buffer. DNA concentration and purity were measured. PCR was performed using primers flanking the FPGS rs1544105 site, and the 316 bp product was purified and sequenced via Sanger sequencing. The PCR primers for genomic DNA are listed in Supplementary Table 11.

#### Homogeneous monoclonal cell screening

Cells with high editing efficiency were expanded to a 12-well plate, digested, gradient diluted, and seeded into a 48-well plate at 1–2 cells per well. After monoclonal colonies formed, the process was repeated with digestion, freezing, DNA extraction, PCR, and sequencing. High-efficiency edited cells were selected for further rounds of screening, ultimately generating the FPGS rs1544105 A > G 293T cell line.

After successful cell line construction, culture supernatants from both WT-293T and Mut-293T cells were tested for mycoplasma contamination using a Mycoplasma Detection Kit (YEASEN, Shanghai, China).

#### qPCR

Total RNA was extracted using pre-chilled Trizol, followed by phenol–chloroform separation and ethanol precipitation. cDNA was synthesized with HiScript III RT SuperMix for qPCR (Vazyme, Nanjing, Jiangsu, China). qPCR was performed with ChamQ SYBR qPCR Master Mix (Vazyme), using ACTIN and GAPDH as internal controls, and specific primers. Relative expression levels were calculated using the 2^-ΔΔCt method. Primer sequences are listed in Supplementary Table 12.

#### WB

Cells were lysed with RIPA buffer containing protease inhibitors for 30 min, and protein concentration was measured by BCA assay. Samples (15 µg) were denatured with 5 × Loading Buffer at 100 °C for 10 min, then subjected to WB using 12% separation gel. Anti-FPGS antibody (ABclonal, #A20973) and anti-β-actin antibody (Servicebio, #GB15001-100) were used as primary antibody and internal control, respectively. Band intensities (Tanon, Shanghai, China) were quantified via densitometry after scanning.

### EMSA

We first employed the transcription factor binding site prediction tool MoLoTool from the HOCOMOCO database to analyze the sequence surrounding rs1544105 and identified putative transcription factors with significant binding potential. Based on approximately 20 bp flanking the mutation site, we designed and synthesized both WT and Mut probes, with a biotin label attached to the 5′ end of the forward strand for chemiluminescent detection (Supplementary Table 13). Nuclear proteins were extracted from WT-293T and Mut-293T cells, and the protein levels of the candidate transcription factor CREB1 were examined by WB (anti-CREB1 antibody, ABclonal, #A1189). Subsequently, EMSA were performed using an EMSA kit (Beyotime, Shanghai, China) with recombinant CREB1 protein (AntibodySystem, #YHD15901) to evaluate the binding activity of CREB1 to DNA fragments harboring the rs1544105 locus.

### Dual-luciferase reporter assay

The FPGS promoter region encompassing the rs1544105 locus, including both WT and Mut sequences, was cloned into the pGL3 luciferase reporter plasmid, while a Renilla luciferase plasmid was used as an internal control (Fig. S12). WT-293T cells were transiently co-transfected with the two plasmids at the indicated ratio using Lipofectamine 2000 (YEASEN). After 24–48 h of incubation, luciferase activity was measured using a dual-luciferase reporter assay kit (YEASEN) and quantified with the IVIS Lumina XR small animal in vivo imaging system (Revvity, Waltham, MA, USA).

### Drug experimental validation

#### Gene expression under drug pressure

MTX (MCE, #HY-14519) at concentrations of 0, 200, and 400 µg/mL was co-incubated with Mut-293T and WT-293T cells for 48 h. Afterward, the qPCR procedure was repeated to assess FPGS gene expression.

#### Extracellular MTX concentration

MTX at concentrations of 200 and 400 µg/mL was co-incubated with Mut-293T and WT-293T cells for 48 h. The supernatant was then collected by centrifugation, concentrated, and analyzed by high-performance liquid chromatography (Shimadzu LC-20AD, Shimadzu, Kyoto, Japan) [25].

LC separation was performed using a C18 column (Ultimate Plus-C18 4.6 × 150 mm, 5 μm, Welch Materials, Shanghai, China), The column temperature was set to 30 °C, with a detection wavelength of 302 nm. The pH of the mobile phase, composed of 0.3% phosphoric acid in water, was adjusted to 2.50 using triethylamine. The mobile phase consisted of a mixture of buffer and acetonitrile in a ratio of 85:15.

#### Cell viability

For CCK8, Cells were seeded onto 96-well plates and treated with MTX at concentrations of 0, 10, 50, 100, 200, and 400 µg/mL, 10 µL per well. After 24 h, 10 µL of CCK8 solution (Vazyme) was added and incubated for 2 h. Cell viability was assessed by measuring the absorbance at 450 nm using a microplate reader (Gen5, BioTek, Winooski, VT, USA).

#### Apoptosis

Cells were seeded in 12-well plates and co-cultured with 0, 400, and 600 µg/mL MTX for 24 h. After collecting the supernatant and cells from each well and centrifuging, the cells were resuspended in Binding Buffer. Annexin V-APC and PI were added and incubated, followed by detection of apoptosis using a BD Canto II flow cytometer (BD Bioscience, San Jose, CA, USA) [60].

#### Animal models

To establish a subcutaneous tumor model using WT-293T and Mut-293T cell lines, the cells were first cultured until they reached approximately 90% confluence. The cells were then harvested and resuspended at a concentration of 2 × 10^7^ cells/mL. A total of 100 μL of the cell suspension was injected into the left axillary fat pad of 6–8-week-old female N-SCID mice (16–18 g). The tumor model was established after two weeks, at which point tumor size and body volume of the mice were measured.

All tumor-bearing mice were closely monitored throughout the experiment. A humane endpoint based on a maximum allowable tumor volume was strictly enforced, and appropriate interventions were promptly taken to minimize animal distress when necessary.

#### Animal experiment

To evaluate the in vivo antitumor efficacy of MTX in WT-293T and Mut-293T xenograft models, MTX was administered intratumorally at a dose of 50 μL of 2 mg/mL per mouse for five consecutive days. Tumor volume and body weight were measured daily using calipers and a digital balance. On day 7, mice were euthanized, and tumor tissues were collected for histological analysis. Tumor sections were prepared and subjected to H&E staining, Ki-67 immunohistochemistry, and TUNEL immunofluorescence assays by Nanjing Youmeng Biotechnology Co., Ltd. (Nanjing, Jiangsu, China). The sections were visualized and imaged using light microscopy.

#### Statistical analysis

All data are presented as mean ± SD from at least three independent experiments. Statistical analysis was conducted using GraphPad Prism 10.2.3 Software (GraphPad Software, San Diego, CA, USA). The specific statistical tests employed for each experiment are detailed in the figure legends [61].

## Supplementary Information

Supplementary Material 1.

## Data Availability

The data supporting the findings of this study can be obtained from the corresponding author upon reasonable request. All supplementary materials and relevant data links are thoroughly cited within the manuscript.
