# Supplementary material for: A single non-coding SNP in FPGS modulates folate drug efficacy in acute lymphoblastic leukemia: data-driven exploration and experimental validation
Source: Mol Biomed. 2025 Nov 21;6:114. doi: 10.1186/s43556-025-00353-9 (PMC12638486; doi:10.1186/s43556-025-00353-9)
Supplement: Supplementary file 1 — Supplementary Material 1. [file 43556_2025_353_MOESM1_ESM.docx]

**Supporting Information**

**A single non-coding SNP in FPGS modulates folate drug efficacy in acute lymphoblastic leukemia: data-driven exploration and experimental validation**

Wenliang Yu ^1,2^ ^†^, Chenyang Li ^1†^, Yuning Meng ^1^, Qiang Li ^1^, Mengyue Gao ^1^, Wei Tang ^1^, Yao Li ^1^, Ziyi Tan ^1^, Xiaoran Zhou ^1^, Zeyang Liu ^1^, Yun Xu ^1^, Zichun Hua ^1-3^*

**Supplementary Table 1.** Main characteristics of eligible studies.

| Author | Year | Ethnicity | Age (years) | Dose of MTX | Source of control | Genotyping method | NOS | Ref |
| --- | --- | --- | --- | --- | --- | --- | --- | --- |
|  |  |  |  |  |  |  |  |  |
| Piwkham | 2015 | Thai | 6.2 (0.4-14)^a^ | NI | PB | TaqMan | 8 | [1] |
| Lautner-Csorba | 2013 | Caucasian | Cases: 5.1 (1-18); controls: 12.5 (1.5-59) | NI | PB | MassARRAY | 9 | [2] |
| Wang | 2014 | Chinese | 7.1 ± 4.0^b^ | 1.5-5 g/m^2^ | Hapmap | TaqMan | 7 | [3] |
| Liu | 2013 | Chinese | 4 (1-15) | 2 g/m^2^ | Hapmap | TaqMan | 7 | [4] |
| Kodidela | 2018 | Indian | Cases: 15.5 ± 10.5; Controls: 24.5 ± 4.8 | NI | PB | TaqMan | 9 | [5] |
| Huang | 2016 | Chinese | Cases: 5.9 ± 4.3; Controls: 3.7 ± 1.6 | High-dose | PB | TaqMan | 8 | [6] |
| Muralidharan | 2019 | Indian | 42.63 ± 0.56 | 10 mg/week to 5 mg every two weeks or max of 25 mg/week | Case^c^ | TaqMan | 8 | [7] |
| Kodidela | 2020 | Indian | 14.5 ± 11.4 | Low-dose | Case | TaqMan | 7 | [8] |
| Moya | 2016 | Caucasian | 51.6 ± 13.4 | 7.5–25 mg/week | Case | TaqMan | 7 | [9] |
| GÓMEZ-GÓMEZ | 2014 | Mexican | Cases: 7.65 ± 4.67; Controls: 9.99 ± 5.49 | NI | PB | TaqMan |  | [10] |
| Liu | 2014 | Chinese | Cases: 4 (1-15) | 2-20 mg/m^2^ per day | 1000 Genomes | MassARRAY |  | [11] |

Note: ^a^: mean (range), ^b^: median ± SD, ^c^: wild type (GG) as control group, MTX: methotrexate, HWE: Hardy-Weinberg equilibrium, NOS: Newcastle-Ottawa assessment scale, ALL: acute lymphocytic leukemia, NI: not information, PB: population-based, AR: rheumatoid arthritis.

**Supplementary Table 2.** SNP data extracted for meta-analysis.

| Author | SNP | No. (case/controls) | Case | | | Control | | | | HWE |
| --- | --- | --- | --- | --- | --- | --- | --- | --- | --- | --- |
|  |  |  | AA | AG | GG | AA | AG | | GG |  |
| Piwkham (2015) | rs1544105 | 95/98 | 42 | 47 | 6 | 58 | 29 | | 11 | 0.0726 |
|  | rs10106 | 95/98 | 6 | 47 | 42 | 12 | 31 | | 55 | 0.1006 |
| Lautner-Csorba (2013) | rs1544105 | 543/529 | 92 | 249 | 202 | 78 | 246 | | 205 | 0.9556 |
|  | rs10106 | 543/529 | 213 | 242 | 88 | 206 | 244 | | 79 | 0.8891 |
| Wang (2014) | rs1544105 | 91/86 | 43 | 34 | 14 | 42 | 34 | | 10 | 0.7477 |
| Liu (2013) | rs1544105 | 164/86 | 75 | 75 | 14 | 42 | 34 | | 10 | 0.7477 |
| Kodidela (2018) | rs1544105 | 149/219 | 31 | 74 | 44 | 27 | 109 | | 83 | 0.6289 |
|  | rs10106 | 145/212 | 49 | 70 | 26 | 82 | 103 | | 27 | 0.8308 |
| Huang (2016) | rs1544105 | 75/31 | 43 | 21 | 11 | 13 | 14 | | 4 | 0.9971 |
| Muralidharan (2019) | rs1544105 | 330/0 | 50 | 157 | 123 | - | - | | - | - |
|  | rs10106† | 64/261 | 17 | 40 | 7 | 103 | 115 | | 43 | 0.5314 |
| Kodidela (2020) | rs1544105 | 73/0 | 15 | 40 | 18 | - | - | | - | - |
| Moya (2016) | rs10106† | 60/134 | 33 | AG+GG: 27 | | 50 | AG+GG: 84 | | | - |
| GÓMEZ-GÓMEZ (2014) | rs10760502 | 70/100 | 19 | 38 | 13 | 66 | 27 | 7 | | 0.2292 |
| Liu (2014) | rs10760502 | 110/103 | 6 | 7 | 97 | 0 | 14 | 89 | | 0.7605 |

Note: †: case group was patients with adverse events, control group was patients without adverse events.

**Supplementary Table 3.** Heterogeneity analysis of the association between *FPGS* SNP polymorphisms and ALL disease progression in the meta-analysis.

| **SNP** | **Genetic comparison model** | **Test of heterogeneity** | |
| --- | --- | --- | --- |
|  |  | **I^2^ (%)** | **P^a^** |
| rs1544105 | AG vs. GG | 70.2 | 0.0004 |
|  | AA vs. GG | 88.2 | < 0.0001 |
|  | AA + AG vs. GG | 82.5 | < 0.0001 |
|  | AA vs. AG + GG | 88.7 | < 0.0001 |
|  | A vs. G | 90.8 | < 0.0001 |
| rs10106 | AG vs. GG | 69.5 | 0.0376 |
|  | AA vs. GG | 0.0 | 0.5099 |
|  | AA + AG vs. GG | 58.3 | 0.0911 |
|  | AA vs. AG + GG | 17.0 | 0.2999 |
|  | A vs. G | 0.0 | 0.3820 |
| rs10760502 | AG vs. GG | 0.0 | 0.4860 |
|  | AA vs. GG | 86.9 | 0.0056 |
|  | AA + AG vs. GG | 53.5 | 0.1424 |
|  | AA vs. AG + GG | 87.1 | 0.0054 |
|  | A vs. G | 90.7 | 0.0010 |

Note: ^a^: P-value of heterogeneity

**Supplementary Table 4.** Heterogeneity analysis of the association between *FPGS* rs1544105 polymorphisms and MTX efficacy index in the meta-analysis.

| **Factor** | **Genetic comparison model** | **Test of heterogeneity** | |
| --- | --- | --- | --- |
|  |  | **I^2^ (%)** | **P^a^** |
| MTX C/D |  |  |  |
| 24 h | AG vs. GG | 0.0 | 0.4273 |
|  | AA vs. GG | 0.0 | 0.4164 |
| 40 h | AG vs. GG | 0.0 | 0.4235 |
|  | AA vs. GG | 38.4 | 0.2024 |
| MTXPGs |  |  |  |
| PG3 | AG vs. GG | 38.4 | 0.2024 |
|  | AA vs. GG | 0.0 | 0.9613 |
| PG4 | AG vs. GG | 0.0 | 0.9613 |
|  | AA vs. GG | 0.0 | 0.9181 |
| PG5 | AG vs. GG | 0.0 | 0.9504 |
|  | AA vs. GG | 0.0 | 0.3843 |

Note: ^a^: P-value of heterogeneity

**Supplementary Table 5.** eQTL query results for rs1544105 in different datasets.

| **Database** | **Data Source** | **Gene** | **P-Value** | **FDR** | **Website link** |
| --- | --- | --- | --- | --- | --- |
| eQTLGen consortium | Significant *cis*-eQTLs | FPGS | 1.1 × 10^-11^ | 0 | https://www.eqtlgen.org/cis-eqtls.html |
| GTExPortal | GTEx Analysis Release V8 (dbGaP Accession phs000424.v8.p2) | FPGS | 3.3 × 10^-15^ to 2.8 × 10^-11^ | 0 | https://www.gtexportal.org/home/testyourown |
| eQTL Catalogue | Open Targets Platform | FPGS | 8.7 × 10^-16^ to 1.1 × 10^-10^ | - | https://platform.opentargets.org/variant/9_127800446_C_T |

**Supplementary Table 6.** STR profiling and genotyping results of WT-293T cells.

|  | **WT-293T (Provided sample)** | | | **HEK-293T (Standard sample)** | | |
| --- | --- | --- | --- | --- | --- | --- |
| **Loci** | **Allele1** | **Allele2** | **Allele3** | **Allele1** | **Allele2** | **Allele3** |
| Yindel |  |  |  |  |  |  |
| AMEL | X | X |  | X | X |  |
| D3S1358 | 15 | 16 | 17 | 15 | 16 | 17 |
| D13S317 | 12 |  |  | 12 |  |  |
| D7S820 | 11 |  |  | 11 |  |  |
| D16S539 | 9 | 13 |  | 9 | 13 |  |
| D8S1179 | 11 | 12 | 14 | 12 | 14 |  |
| Penta D | 9 | 10 |  | 9 | 10 |  |
| D19S433 | 18 |  |  | 18 |  |  |
| D5S818 | 8 |  |  | 8 | 9 |  |
| D21S11 | 28 |  |  | 28 | 30.2 |  |
| TPOX | 11 |  |  | 11 |  |  |
| D1S1656 | 15 | 17.3 |  |  |  |  |
| D6S1043 | 11 |  |  |  |  |  |
| D2S441 | 11 | 15 |  |  |  |  |
| D12S391 | 19 | 21 |  |  |  |  |
| D2S1338 | 19 |  |  | 19 |  |  |
| vWA | 16 | 19 |  | 16 | 19 |  |
| Penta E | 7 | 15 |  | 7 | 15 |  |
| TH01 | 7 | 9.3 |  | 7 | 9.3 |  |
| D18S51 | 17 | 18 |  | 17 | 18 |  |
| CSF1PO | 11 | 12 |  | 11 | 12 |  |
| FGA | 23 |  |  | 23 |  |  |

**Supplementary Table 7.** PRISMA checklist.

| **Section/topic** | **#** | **Checklist item** | **Reported on page #** |
| --- | --- | --- | --- |
| **TITLE** | | |  |
| Title | 1 | Identify the report as a systematic review, meta-analysis, or both. | 1 |
| **ABSTRACT** | | |  |
| Structured summary | 2 | Provide a structured summary including, as applicable: background; objectives; data sources; study eligibility criteria, participants, and interventions; study appraisal and synthesis methods; results; limitations; conclusions and implications of key findings; systematic review registration number. | 2 |
| **INTRODUCTION** | | |  |
| Rationale | 3 | Describe the rationale for the review in the context of what is already known. | 3, 4 |
| Objectives | 4 | Provide an explicit statement of questions being addressed with reference to participants, interventions, comparisons, outcomes, and study design (PICOS). | 4 |
| **METHODS** | | |  |
| Protocol and registration | 5 | Indicate if a review protocol exists, if and where it can be accessed (e.g., Web address), and, if available, provide registration information including registration number. | 14 |
| Eligibility criteria | 6 | Specify study characteristics (e.g., PICOS, length of follow-up) and report characteristics (e.g., years considered, language, publication status) used as criteria for eligibility, giving rationale. | 14 |
| Information sources | 7 | Describe all information sources (e.g., databases with dates of coverage, contact with study authors to identify additional studies) in the search and date last searched. | 14 |
| Search | 8 | Present full electronic search strategy for at least one database, including any limits used, such that it could be repeated. | Supplementary Table 8 |
| Study selection | 9 | State the process for selecting studies (i.e., screening, eligibility, included in systematic review, and, if applicable, included in the meta-analysis). | 14 |
| Data collection process | 10 | Describe method of data extraction from reports (e.g., piloted forms, independently, in duplicate) and any processes for obtaining and confirming data from investigators. | 15 |
| Data items | 11 | List and define all variables for which data were sought (e.g., PICOS, funding sources) and any assumptions and simplifications made. | 15 |
| Risk of bias in individual studies | 12 | Describe methods used for assessing risk of bias of individual studies (including specification of whether this was done at the study or outcome level), and how this information is to be used in any data synthesis. | 15 |
| Summary measures | 13 | State the principal summary measures (e.g., risk ratio, difference in means). | 15, 16 |
| Synthesis of results | 14 | Describe the methods of handling data and combining results of studies, if done, including measures of consistency (e.g., I^2^) for each meta-analysis. | 15, 16 |

Page 1 of 2

| **Section/topic** | **#** | **Checklist item** | **Reported on page #** |
| --- | --- | --- | --- |
| Risk of bias across studies | 15 | Specify any assessment of risk of bias that may affect the cumulative evidence (e.g., publication bias, selective reporting within studies). | 15, 16 |
| Additional analyses | 16 | Describe methods of additional analyses (e.g., sensitivity or subgroup analyses, meta-regression), if done, indicating which were pre-specified. | 15, 16 |
| **RESULTS** | | |  |
| Study selection | 17 | Give numbers of studies screened, assessed for eligibility, and included in the review, with reasons for exclusions at each stage, ideally with a flow diagram. | 5  Fig. 1b |
| Study characteristics | 18 | For each study, present characteristics for which data were extracted (e.g., study size, PICOS, follow-up period) and provide the citations. | 5  Supplementary Table 1, 2 |
| Risk of bias within studies | 19 | Present data on risk of bias of each study and, if available, any outcome level assessment (see item 12). | 5, 6  Fig. 1c |
| Results of individual studies | 20 | For all outcomes considered (benefits or harms), present, for each study: (a) simple summary data for each intervention group (b) effect estimates and confidence intervals, ideally with a forest plot. | 5, 6  Fig. 2, 3 |
| Synthesis of results | 21 | Present results of each meta-analysis done, including confidence intervals and measures of consistency. | 5, 6  Fig. 2, 3 |
| Risk of bias across studies | 22 | Present results of any assessment of risk of bias across studies (see Item 15). | 6  Fig. S1, S2 |
| Additional analysis | 23 | Give results of additional analyses, if done (e.g., sensitivity or subgroup analyses, meta-regression [see Item 16]). | 5, 6  Fig. S3, S4 |
| **DISCUSSION** | | |  |
| Summary of evidence | 24 | Summarize the main findings including the strength of evidence for each main outcome; consider their relevance to key groups (e.g., healthcare providers, users, and policy makers). | 10-12 |
| Limitations | 25 | Discuss limitations at study and outcome level (e.g., risk of bias), and at review-level (e.g., incomplete retrieval of identified research, reporting bias). | 13 |
| Conclusions | 26 | Provide a general interpretation of the results in the context of other evidence, and implications for future research. | 14 |
| **FUNDING** | | |  |
| Funding | 27 | Describe sources of funding for the systematic review and other support (e.g., supply of data); role of funders for the systematic review. | 23 |

*From:*  Moher D, Liberati A, Tetzlaff J, Altman DG, The PRISMA Group (2009). Preferred Reporting Items for Systematic Reviews and Meta-Analyses: The PRISMA Statement. PLoS Med 6(7): e1000097. doi:10.1371/journal.pmed1000097

For more information, visit: **www.prisma-statement.org**.

Page 2 of 2

**Supplementary Table 8.** Search strategies.

| **Databases** | **Search strategies or search terms** |
| --- | --- |
| Embase | #1 'leukemia'/exp OR 'leukemia'  #2 'acute lymphoblastic leukemia'  #3 #1 OR #2  #4 'mutation'  #5 'variant'  #6 'polymorphism'  #7 #4 OR #5 OR #6  #8 'folate'  #9 'fpgs gene'  #10 'folylpolyglutamate synthase'  #11 #8 OR #9 OR #10  #12 #3 AND #7 AND #11 AND [humans]/lim AND [embase]/lim |
| PubMed | #1 ("leukaemia"[All Fields] OR "leukemia"[MeSH Terms] OR "leukemia"[All Fields] OR "leukaemias"[All Fields] OR "leukemias"[All Fields] OR "leukemia s"[All Fields] OR ("acute lymphoblastic leukaemia"[All Fields] OR "precursor cell lymphoblastic leukemia lymphoma"[MeSH Terms] OR ("precursor"[All Fields] AND "cell"[All Fields] AND "lymphoblastic"[All Fields] AND "leukemia lymphoma"[All Fields]) OR "precursor cell lymphoblastic leukemia lymphoma"[All Fields] OR ("acute"[All Fields] AND "lymphoblastic"[All Fields] AND "leukemia"[All Fields]) OR "acute lymphoblastic leukemia"[All Fields])) AND ("polymorphic"[All Fields] OR "polymorphics"[All Fields] OR "polymorphism s"[All Fields] OR "polymorphism, genetic"[MeSH Terms] OR ("polymorphism"[All Fields] AND "genetic"[All Fields]) OR "genetic polymorphism"[All Fields] OR "polymorphism"[All Fields] OR "polymorphisms"[All Fields] OR ("mutate"[All Fields] OR "mutated"[All Fields] OR "mutates"[All Fields] OR "mutating"[All Fields] OR "mutation"[MeSH Terms] OR "mutation"[All Fields] OR "mutations"[All Fields] OR "mutation s"[All Fields] OR "mutational"[All Fields] OR "mutator"[All Fields] OR "mutators"[All Fields]) OR ("variant"[All Fields] OR "variant s"[All Fields] OR "variants"[All Fields])) AND ("folic acid"[MeSH Terms] OR ("folic"[All Fields] AND "acid"[All Fields]) OR "folic acid"[All Fields] OR "folate"[All Fields] OR "folates"[All Fields] OR "FPGS"[All Fields] OR ("folylpolyglutamate synthetase"[Supplementary Concept] OR "folylpolyglutamate synthetase"[All Fields] OR "folylpolyglutamate synthase"[All Fields])) |
| Medline | #1 'leukemia'/exp OR 'leukemia'  #2 'acute lymphoblastic leukemia'  #3 #1 OR #2  #4 'mutation'  #5 'variant'  #6 'polymorphism'  #7 #4 OR #5 OR #6  #8 'folate'  #9 'fpgs gene'  #10 'folylpolyglutamate synthase'  #11 #8 OR #9 OR #10  #12 #3 AND #7 AND #11 AND [humans]/lim AND [medline]/lim |
| Wanfang | (leukemia or acute lymphoblastic leukemia) and (mutation or variant or polymorphism) and (folate or FPGS or folylpolyglutamate synthase) |
| CNKI | (SU='leukemia' or SU='acute lymphoblastic leukemia') and (SU='mutation' or SU='variant' or SU='polymorphism') and (SU='folate' or SU='FPGS' or SU='folylpolyglutamate synthase') and Academic journals |

**Supplementary Table 9.** The sequences of oligonucleotides used in this study.

| **Name** | **Sequence (5’-3’)** |
| --- | --- |
| FPGS_Spacer_fwd | CACCGCCTTCAGTGTTTTGCTGCTAGTTTA |
| FPGS_Spacer_rev | CTCTTAAACTAGCAGCAAAACACTGAAGGC |
| scaffold_fwd | AGAGCTAGAAATAGCAAGTTAAAATAAGGCTAGTCCGTTATCAACTTGAAAAAGTGGCACCGAGTCG |
| scaffold_rev | GCACCGACTCGGTGCCACTTTTTCAAGTTGATAACGGACTAGCCTTATTTTAACTTGCTATTTCTAG |
| FPGS_19AtoG_q1_fwd | GTGCAATCAAGCCCTTCGACGTAAACGCATATCCATAGCAGCAAAACACTGCCATACTT |
| FPGS_19AtoG_q1_rev | CGCGAAGTATGGCAGTGTTTTGCTGCTATGGATATGCGTTTACGTCGAAGGGCTTGATT |
| FPGS_19AtoG_not_fwd | GTGCAATCAAGCCCTTCGACGTAAACGCATATCCATAGCAGCAAAACACTGCCTTATCTGGG |
| FPGS_19AtoG_not_rev | CTGACCCAGATAAGGCAGTGTTTTGCTGCTATGGATATGCGTTTACGTCGAAGGGCTTGATT |

**Supplementary Table 10.** The sequences of epegRNA construction verification primers.

| **Target site** | **Sequence (5’-3’)** |
| --- | --- |
| epegRNA_F | GAGGGCCTATTTCCCATGAT |
| epegRNA_R | TATGGAAAAACGCCAGCAAC |

**Supplementary Table 11.** The sequences of PCR primers for genomic DNA.

| **Name** | **Sequence (5’-3’)** |
| --- | --- |
| FPGS_ rs1544105_genomic_fwd | CTCCTTCCCTGTCTCCCCAG |
| FPGS_ rs1544105_genomic_rev | GAGATGGTTCTCTGCACCCT |

**Supplementary Table 12.** The sequences of specific qPCR primers and internal control primers.

| **Name** | **Sequence (5’-3’)** |
| --- | --- |
| FPGS | F-ATACCCTGCAGACCAATGCC |
|  | R-CTCCGTGCCAGGTACAGTTC |
| CDK9 | F-CCATTACAGCCTTGCGGGAGAT |
|  | R-CAGCAAGGTCATGCTCGCAGAA |
| SH2D3C | F-CCTTCAGCAGTGGAGGTAGACC |
|  | R-GGAGAACTTCACATAGTCGCTGC |
| TOR2A | F-CTTTTCTCCCGTCCTCACTTC |
|  | R-GGGCATCTTGTCCATCTCATCG |
| Eng | F-CGGTGGTCAATATCCTGTCGAG |
|  | R-AGGAAGTGTGGGCTGAGGTAGA |
| AK1 | F-GCAAGGAGAAGAGTTTGAGCGAC |
|  | R-GATGGTCTCCTCATTGTCGTCC |
| ST6GALNAC6 | F-TGAGGTCTTCCATTACGGCTCC |
|  | R-CTGCTGACAATCACACACTGGTG |
| ACTB | F-CTCTTCCAGCCTTCCTTCCT |
|  | R-AGCACTGTGTTGGCGTACA |
| GADPH | F-TGCACCACCAACTGCTTAGC |
|  | R-GGCATGGACTGTGGTCATGAG |

**Supplementary Table 13.** EMSA probe sequences.

| **Name** | **Sequence** |
| --- | --- |
| WT probe | F: 5′-AAGCCCTTCGATGTAAACGCATAT-3′ |
|  | R: 3′-TTCGGGAAGCTACATTTGCGTATA-5′ |
| Mut probe | F: 5′-AAGCCCTTCGACGTAAACGCATAT-3′ |
|  | R: 3′-TTCGGGAAGCTGCATTTGCGTATA-5′ |

**
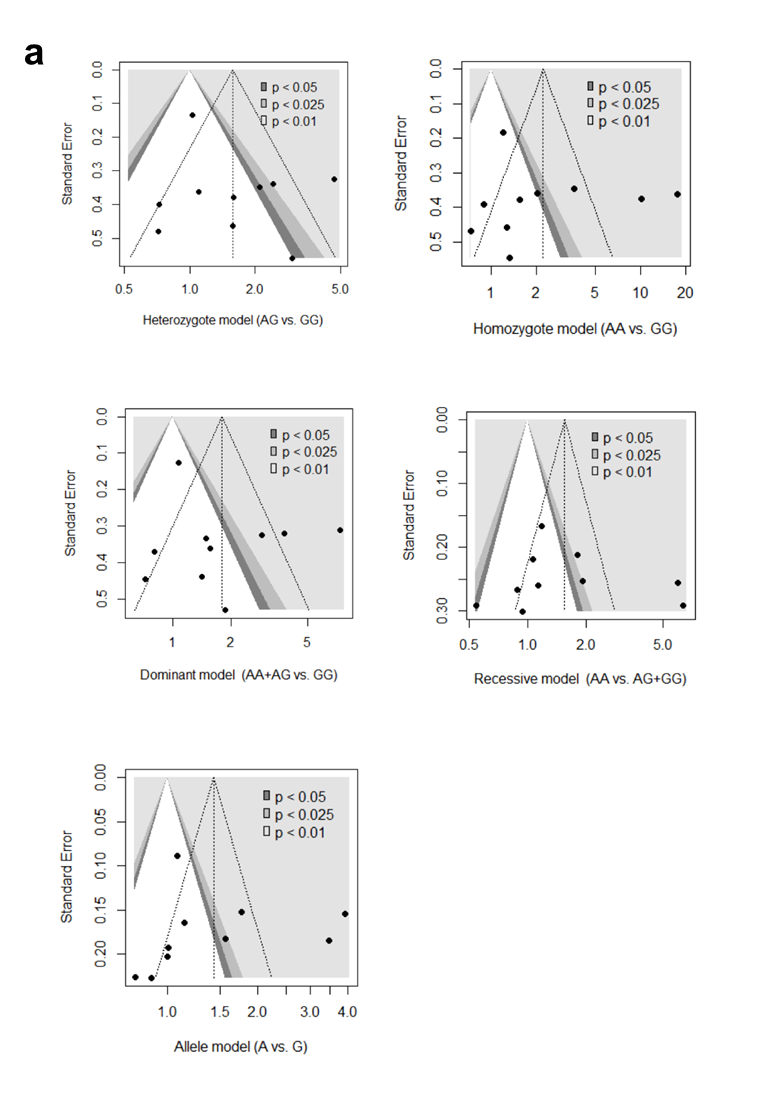
**

**Fig. S1** *Cont.*

**
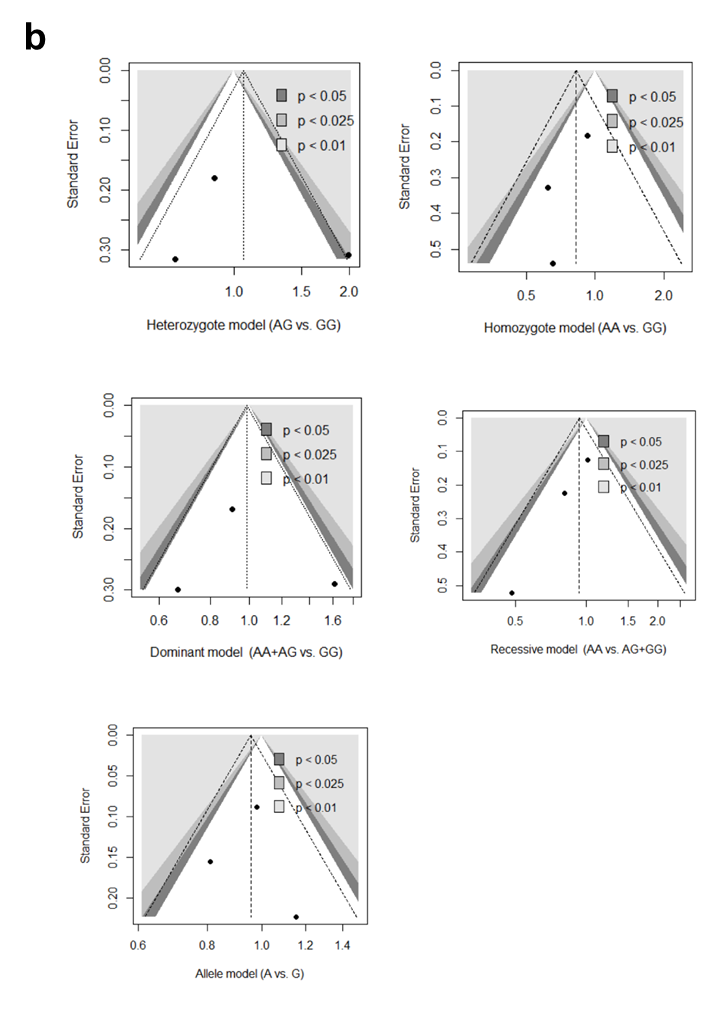
**

**Fig. S1** *Cont.*

**
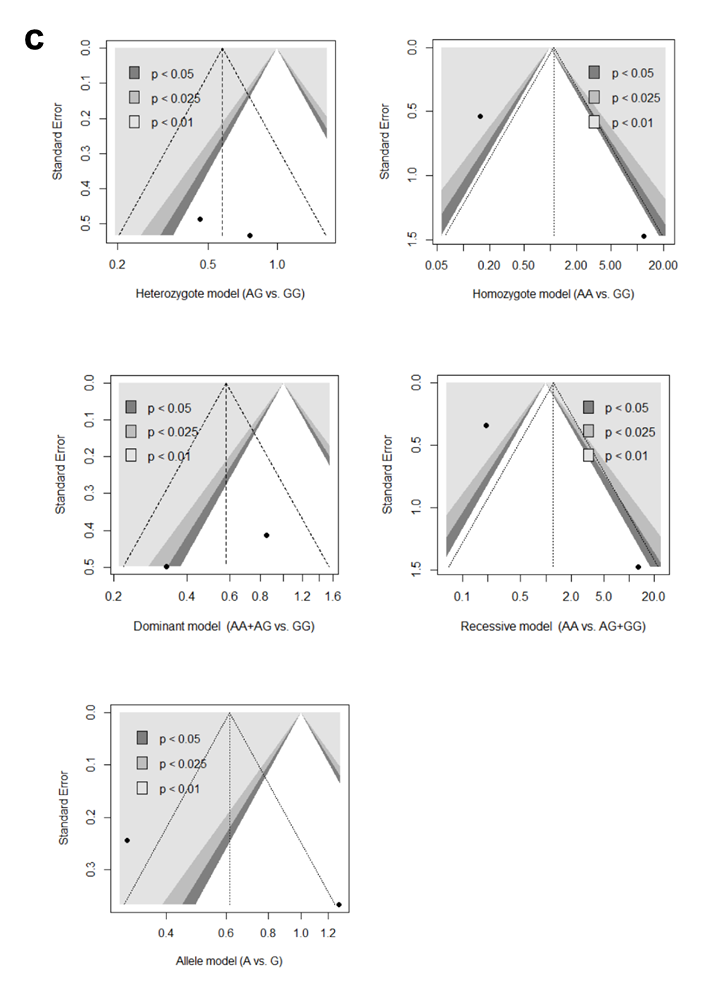
**

**Fig. S1** Funnel plot of FPGS SNPs polymorphisms and ALL disease progression rate. **a** rs1544105. **b** rs10106. **c** rs10760502.

**
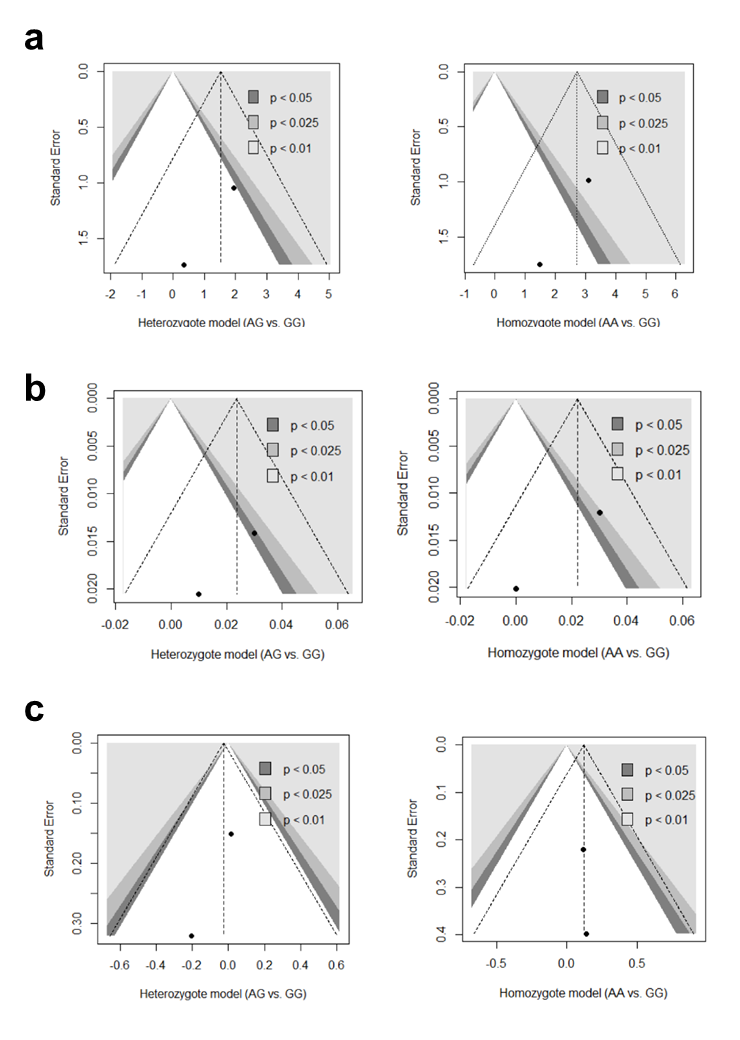
**

**Fig. S2** *Cont.*

**
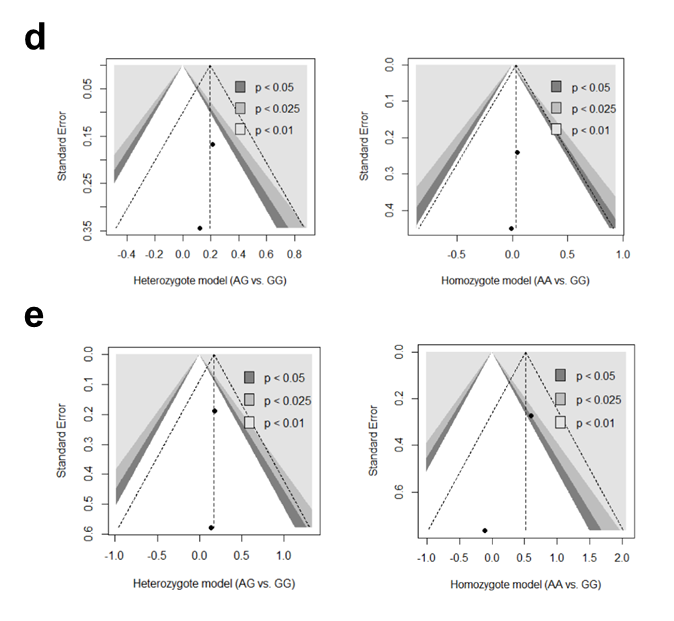
**

**Fig S2** Funnel plot of FPGS rs1544105 polymorphisms and MTX-related clinical indices following administration. **a** 24h MTX C/D. **b** 40h MTX C/D. **c** MTXPG3. **d** MTXPG4. **e** MTXPG5.

**
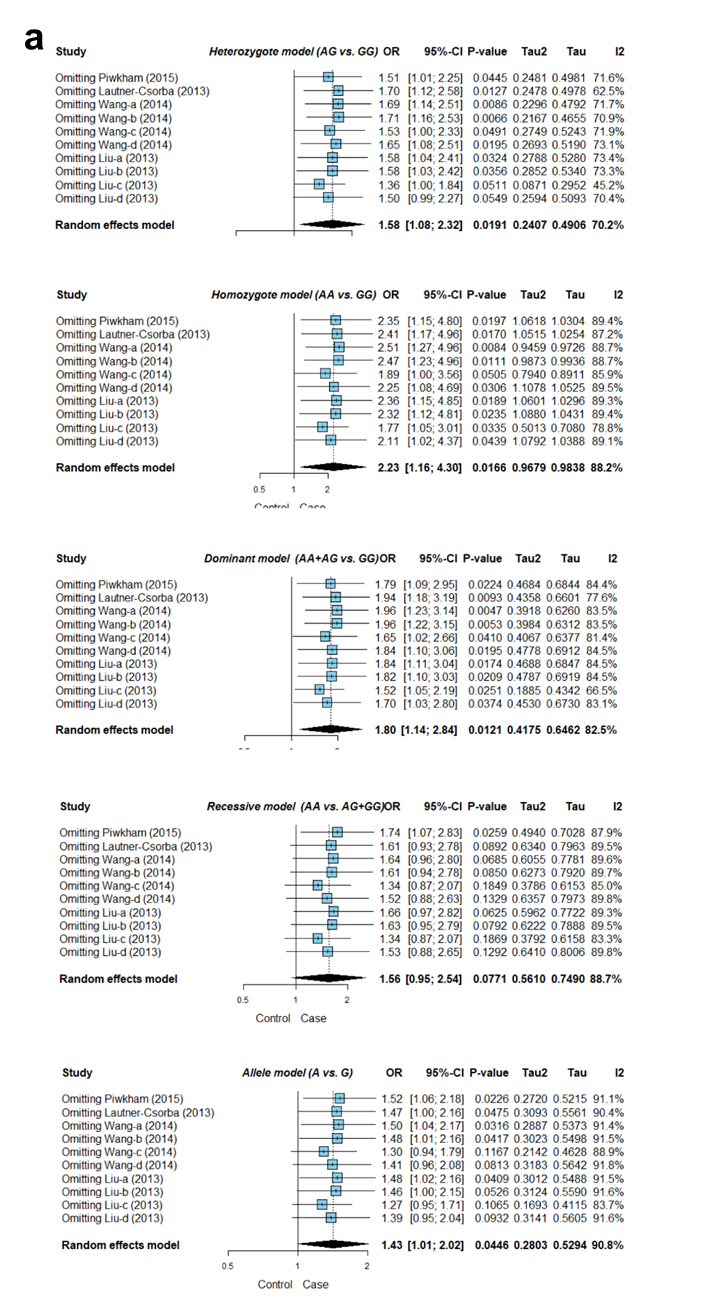
**

**Fig. S3** *Cont.*

**
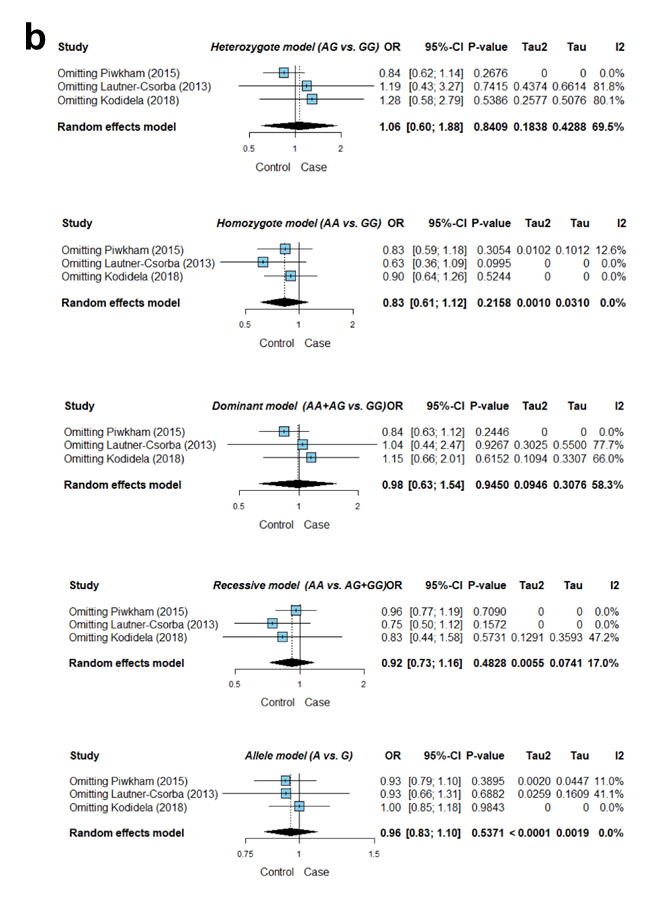
**

**Fig. S3** *Cont.*

**
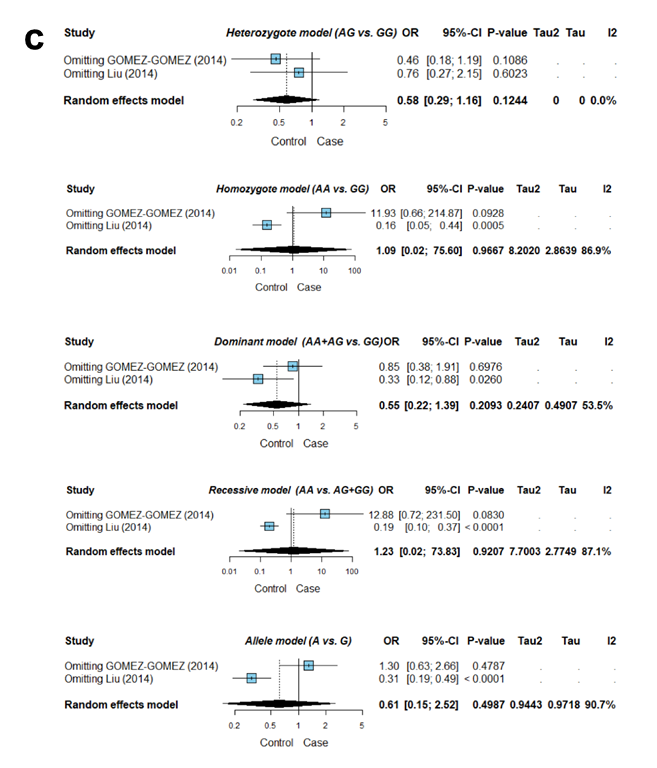
**

**Fig. S3** Forest plot for sensitivity analysis of FPGS SNPs polymorphisms and ALL disease progression rate. **a** rs1544105. **b** rs10106. **c** rs10760502.

**
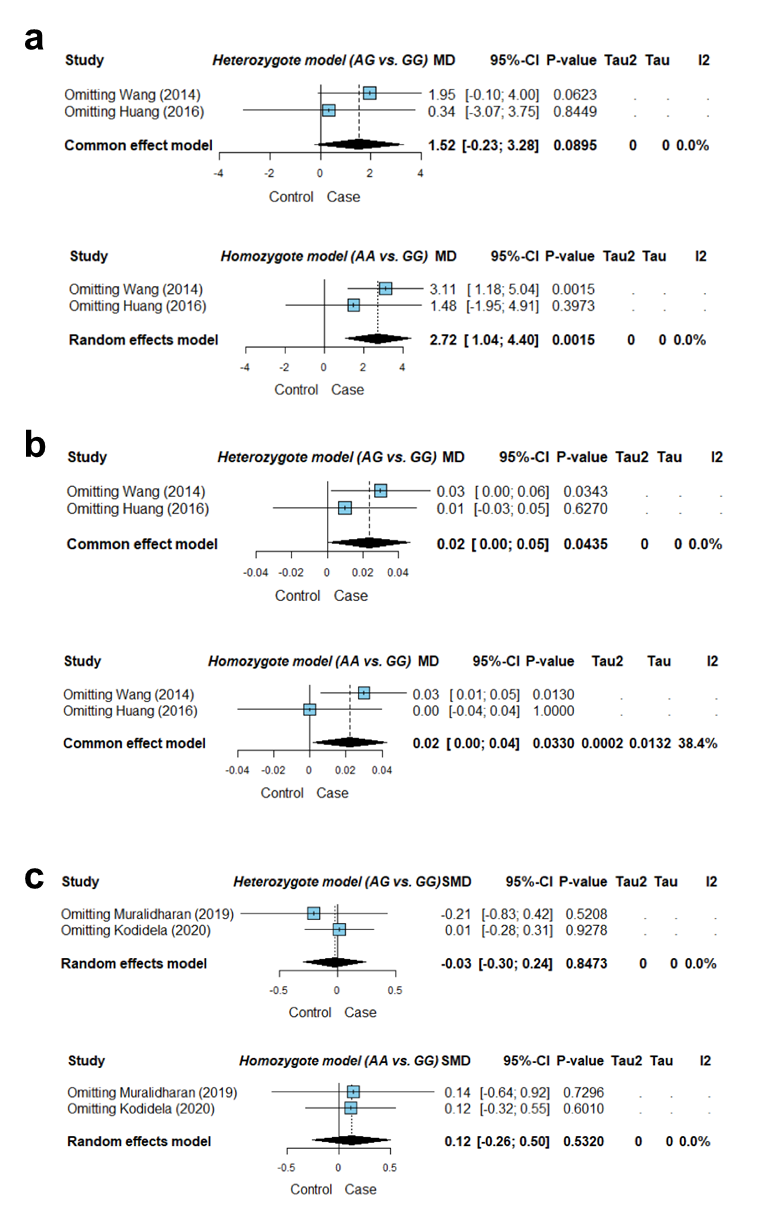
**

**Fig. S4** *Cont.*

**
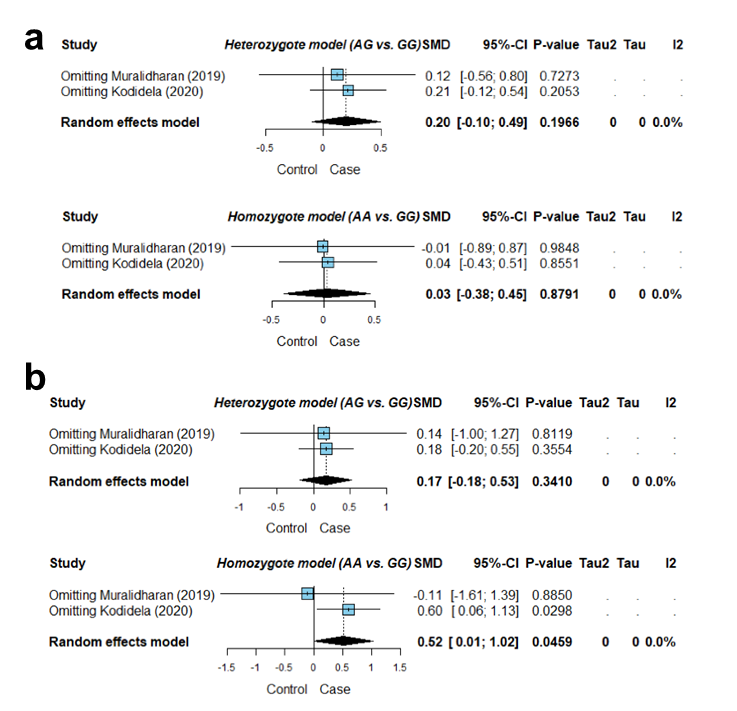
**

**Fig. S4** Forest plot for sensitivity analysis of FPGS rs1544105 polymorphisms and MTX-related clinical indices following administration. **a** 24h MTX C/D. **b** 40h MTX C/D. **c** MTXPG3. **d** MTXPG4. **e** MTXPG5.


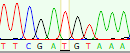


**Fig. S5** Sequencing results of *FPGS* rs1544105 locus in WT-293T cells.

**
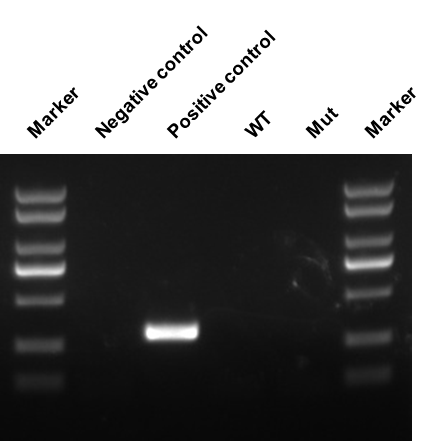
**

**Fig. S6** Mycoplasma detection results of WT-293T and Mut-293T cell cultures.

**
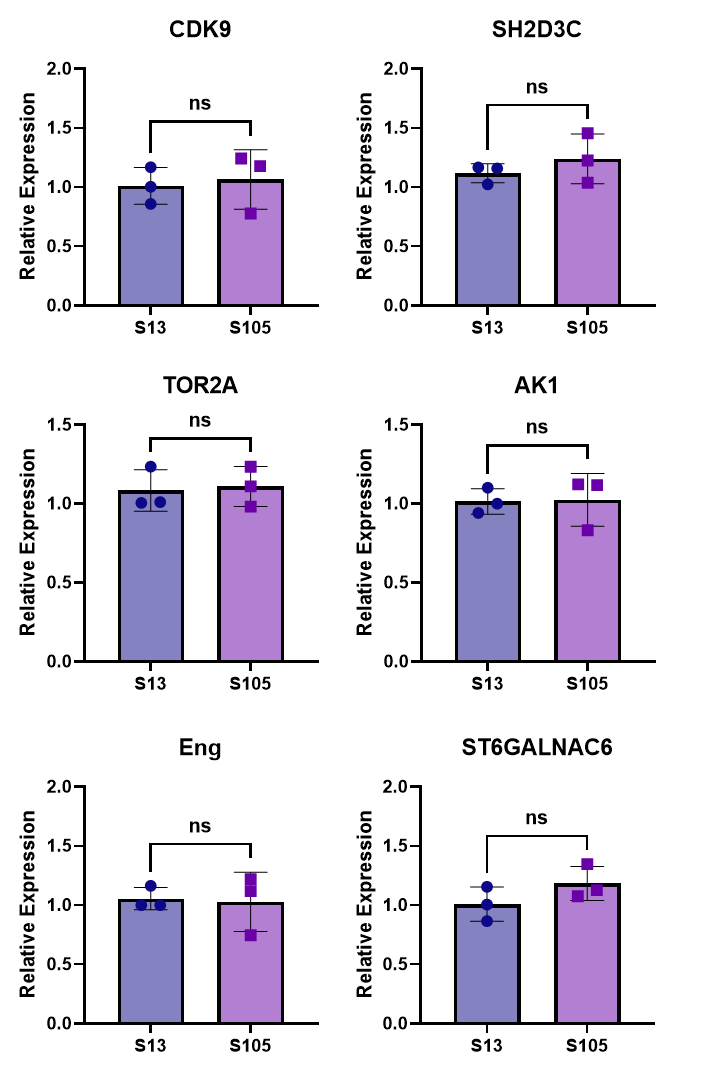
**

**Fig. S7** Quantitative comparison of mRNA levels of the three upstream and three downstream genes of FPGS in WT-293T and mut-293T cells.


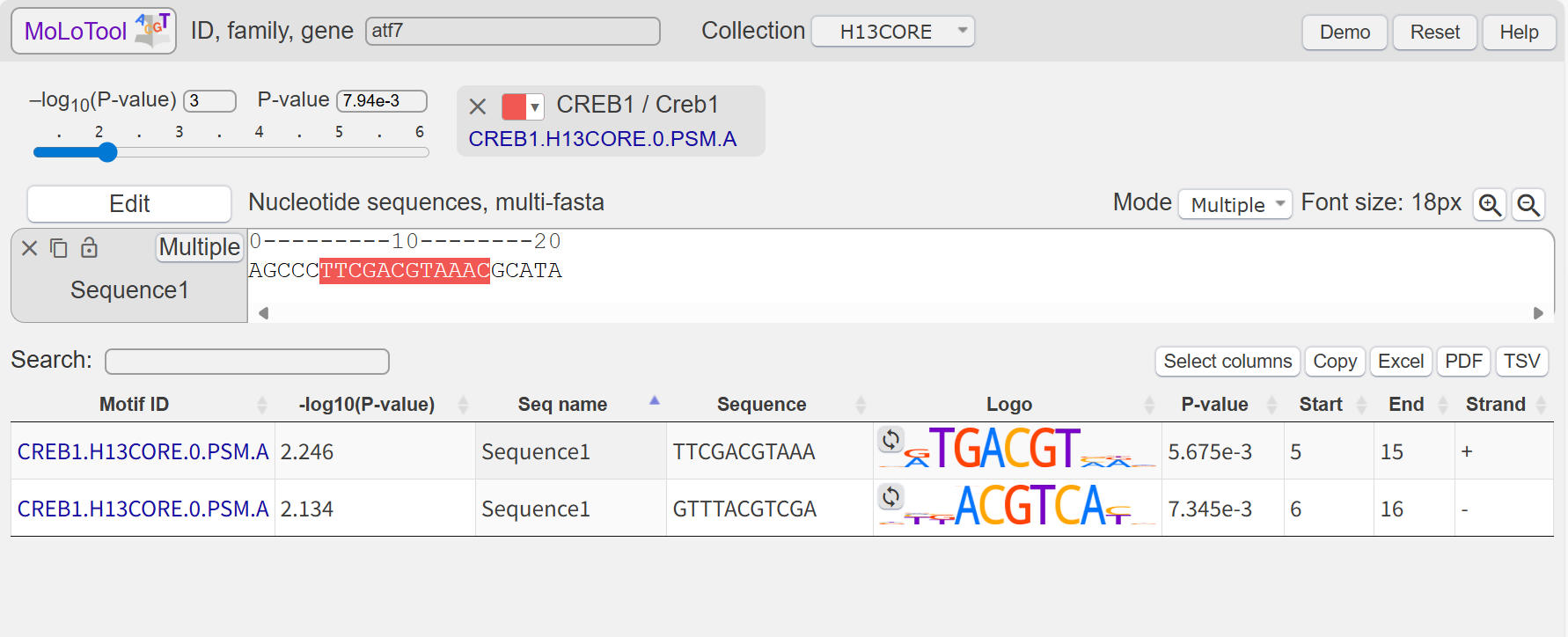


**Fig. S8** Transcription factor prediction for the sequence harboring rs1544105 using the MoLoTool platform.

**
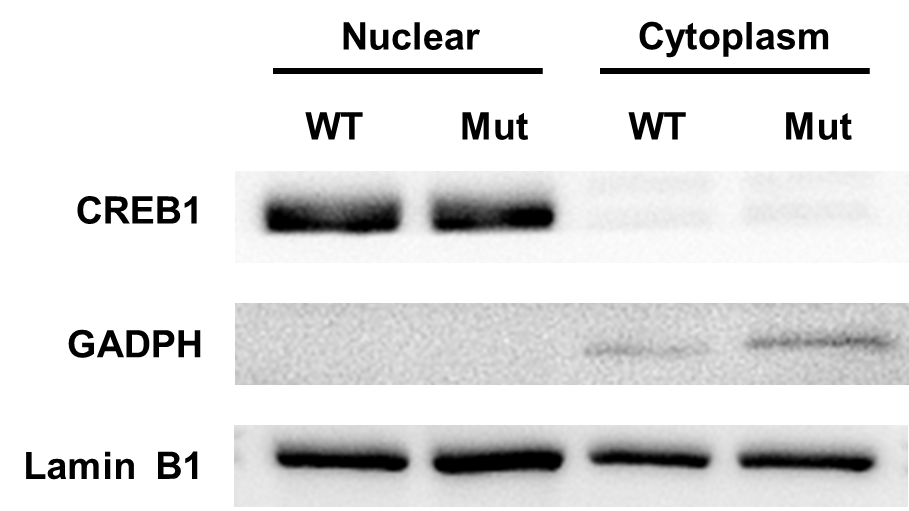
**

**Fig. S9** Western blot analysis of CREB1 in nuclear extracts from WT-293T and Mut-293T cells.

**
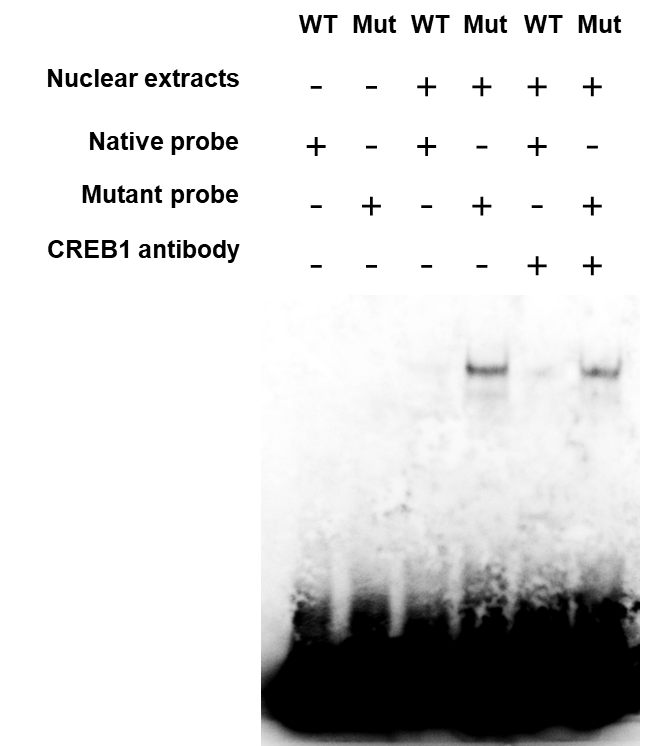
**

**Fig. S10** EMSA results using recombinant CREB1 protein.

**
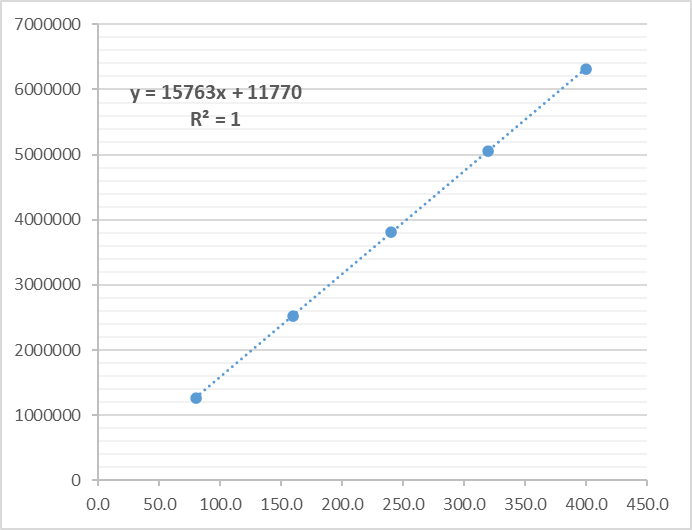
**

**Fig. S11** Standard curve for quantification of HPLC peak area.

**
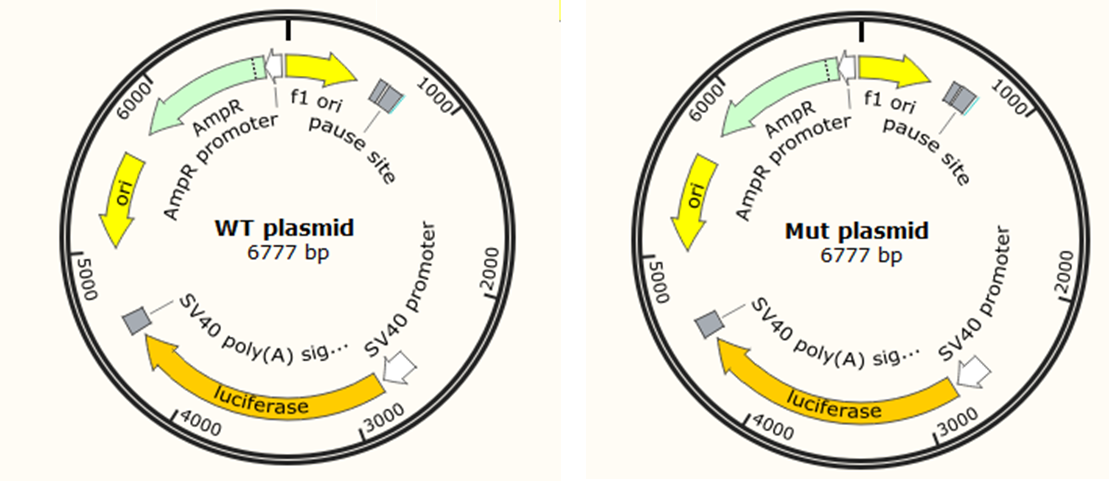
**

**Fig. S12** Schematic maps of the dual-luciferase reporter plasmid used in the assay.

**Supplementary references**

[1] Piwkham D, Siriboonpiputtana T, Beuten J, Pakakasama S, Gelfond J A, Paisooksantivatana K, et al. Mutation Screening and Association Study of the Folylpolyglutamate Synthetase (FPGS) Gene with Susceptibility to Childhood Acute Lymphoblastic Leukemia. Asian Pac J Cancer Prev. 2015;16(11):4727-32. <https://doi.org/10.7314/apjcp.2015.16.11.4727>.

[2] Lautner-Csorba O, Gezsi A, Erdelyi D J, Hullam G, Antal P, Semsei A F, et al. Roles of genetic polymorphisms in the folate pathway in childhood acute lymphoblastic leukemia evaluated by Bayesian relevance and effect size analysis. PLoS One. 2013;8(8):e69843. <https://doi.org/10.1371/journal.pone.0069843>.

[3] Wang S M, Sun L L, Zeng W X, Wu W S, Zhang G L. Influence of genetic polymorphisms of FPGS, GGH, and MTHFR on serum methotrexate levels in Chinese children with acute lymphoblastic leukemia. Cancer Chemother Pharmacol. 2014;74(2):283-9. <https://doi.org/10.1007/s00280-014-2507-8>.

[4] Liu S G, Gao C, Zhang R D, Jiao Y, Cui L, Li W J, et al. FPGS rs1544105 polymorphism is associated with treatment outcome in pediatric B-cell precursor acute lymphoblastic leukemia. Cancer Cell Int. 2013;13(1):107. <https://doi.org/10.1186/1475-2867-13-107>.

[5] Kodidela S, Pradhan S C, Dubashi B, Basu D. Interethnic differences in single and haplotype structures of folylpolyglutamate synthase and gamma-glutamyl hydrolase variants and their influence on disease susceptibility to acute lymphoblastic leukemia in the Indian population: An exploratory study. Indian Journal of Medical and Paediatric Oncology. 2018;39(03):331-38.

[6] Huang Z, Tong H F, Li Y, Qian J C, Wang J X, Wang Z, et al. Effect of the Polymorphism of Folylpolyglutamate Synthetase on Treatment of High-Dose Methotrexate in Pediatric Patients with Acute Lymphocytic Leukemia. Med Sci Monit. 2016;22(4967-73. <https://doi.org/10.12659/msm.899021>.

[7] Muralidharan N, Sundaram R, Kodidela S, Chengappa K G, Mariaselvam C M, Misra D P, et al. Folyl polyglutamate synthethase (FPGS) gene polymorphisms may influence methotrexate adverse events in South Indian Tamil Rheumatoid Arthritis patients. Pharmacogenomics J. 2020;20(2):342-49. <https://doi.org/10.1038/s41397-019-0097-x>.

[8] Kodidela S, Dorababu P, Thakkar D N, Dubashi B, Sundaram R, Muralidharan N, et al. Association of NUDT15*3 and FPGS 2572C>T Variants with the Risk of Early Hematologic Toxicity During 6-MP and Low-Dose Methotrexate-Based Maintenance Therapy in Indian Patients with Acute Lymphoblastic Leukemia. Genes (Basel). 2020;11(6):<https://doi.org/10.3390/genes11060594>.

[9] Moya P, Salazar J, Arranz M J, Díaz-Torné C, del Río E, Casademont J, et al. Methotrexate pharmacokinetic genetic variants are associated with outcome in rheumatoid arthritis patients. Pharmacogenomics. 2016;17(1):25-9. <https://doi.org/10.2217/pgs.15.150>.

[10] Gómez-Gómez Y, Organista-Nava J, Rangel-Rodriguez C A, Illades-Aguiar B, Moreno-Godínez M E, Alarcón-Romero L D, et al. Effect of folylpolyglutamate synthase A22G polymorphism on the risk and survival of patients with acute lymphoblastic leukemia. Oncol Lett. 2014;8(2):731-35. <https://doi.org/10.3892/ol.2014.2175>.

[11] Liu S G, Gao C, Li Z G, Li W J, Cui L, Zhao X X, et al. [Correlation analysis of FPGS rs10760502G>a polymorphism with prognosis and MTX-related toxicity in pediatric B-cell acute lymphoblastic leukemia]. Zhongguo Shi Yan Xue Ye Xue Za Zhi. 2014;22(2):291-7. <https://doi.org/10.7534/j.issn.1009-2137.2014.02.006>.
